# A biaxial tensional model for early vertebrate morphogenesis

**DOI:** 10.1140/epje/s10189-022-00184-4

**Published:** 2022-04-08

**Authors:** Vincent Fleury, Anick Abourachid

**Affiliations:** 1 Laboratoire MSC, CNRS/Universit é de Paris Cité, UMR 7057, 10 rue Alice Domont et Ĺeonie Duquet, 75013 Paris, France; 2 Laboratoire Mécanismes Adaptatifs et Evolution, UMR 7179 MNHN/CNRS, CP 55, 57 rue Cuvier, 75231 Paris Cedex 05, France

## Abstract

**Abstract:**

We propose a simple biaxial tensional model which is able to reproduce at a qualitative level several aspects of early stages of vertebrate morphogenesis. The model is based on subsequent excitable contractions of an orthoradial and periclinal (radial) set of contracting lines, which generate first the basic embryonic pattern (a motile tube), and second the lateral orifices such as ears, eyes, mouth, gills, etc. An important aspect of the model is the self-arresting character of the process, akin to wound healing. At later stages, the biaxial lines may also work in extension, and this generates a developmental feedback which is quadratic with respect to curvature.

**Graphic abstract:**

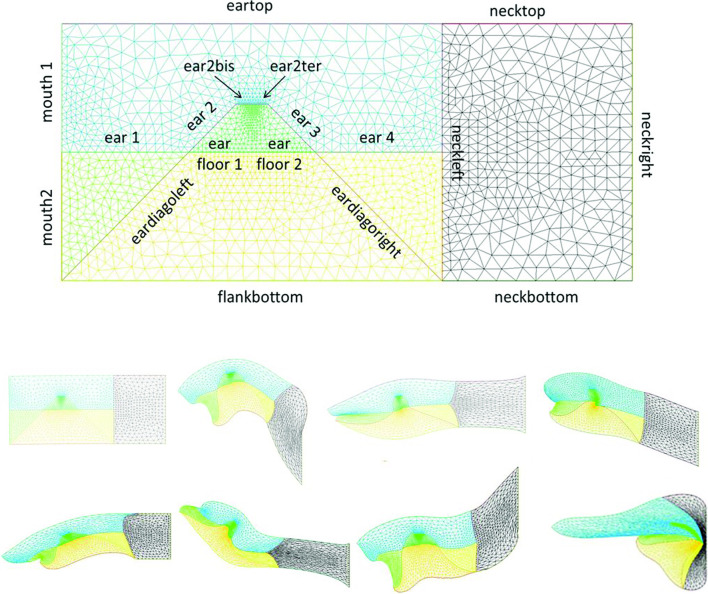

**Supplementary Information:**

The online version contains supplementary material available at 10.1140/epje/s10189-022-00184-4.

## Introduction

### General background

It is often believed that embryogenesis arises from development and growth of standing patterns of biochemicals *à la Turing *[1]. However, in recent years, physical forces have been invoked in many developmental processes, either as influencing indirectly embryogenesis by mechanisms called mechano-transduction (under compressive forces [2] or shear forces [3]) or directly by general conservation physical laws which constrain the development [4]. Our main purpose in this article is to address in a simple model the origin and effects of the pattern of forces which give rise to the fundamentals of the vertebrate body structure.

### Pattern of forces driving embryogenesis: rings

Concerning the force that drives vertebrate embryogenesis, this question has been greatly simplified by the recent discovery of the crucial role of contractile rings [5–7]. Indeed, in the specific case of vertebrate development, it has been found that early embryogenesis arises by tension forces exerted by aligned cells forming “belts” or “purse-strings” around the blastula [5, 7, 8]. These “purse-strings” are akin to very small proto-muscles. While the initial reports found that gastrulation was a viscous flow driven by one single peripheral ring [5], it was found that there exist other rings corresponding to nested rings of cellular cleavage inside the blastodisc [8–10]. The discovery of these rings adds up to one extra-embryonic ring which was already known, the ring forming the amniotic sac [6, 11]. These “purse-strings” are found at the earliest stages on the reference configuration for embryogenesis which, in the avian case is a disc (the “ blastodisc ” [12]). The origin of these rings is in the boundary condition of the oocyte, and the dichotomous nature of oocyte cleavage. Repeated cleavage generates differentiated rings [13]. The contraction of these “purse-strings”, akin to myogenic contraction [14], reshuffles and folds the embryonic disc in a deterministic fashion [5, 7]. This view has been confirmed recently [15].

The view of embryogenesis which has arisen lately is therefore that of a visco-elastic deformation (viscous in the long run) of a reference configuration which is a disc formed of a set of encased rings, which contract. The fundamental structure of a vertebrate is the deformed configuration, or attractor, starting from such a reference configuration : a set of encased tubes, like Russian dolls (the amnion and chorion, the body wall, the neural and digestive tubes, the urogenital tract). This fundamental plan stems from the fact that when encased or nested, soft, viscous, planar, rings contract, the deformed configuration is a set of 3D encased or nested tubes [8, 9].

**Fig. 1:**
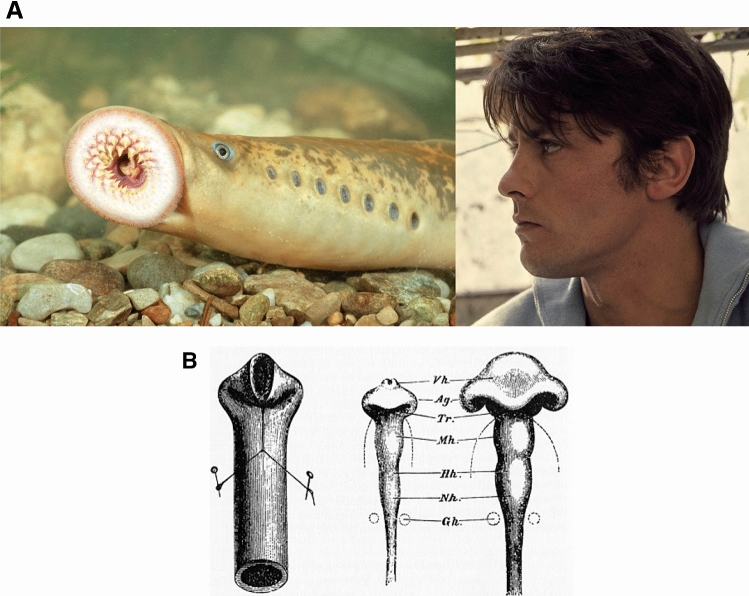
**a** To the left, the lamprey or “flute-fish” exhibits a striking structure composed of a cylindrical body, having cylindrical “holes” with translational symmetry along the dorso-ventral boundary, and one large anterior circle (the mouth), located around the axis of symmetry. To the right, a typical human profile. In higher vertebrates, head flexure locates the mouth, nose, eyes and ears along a deformed anterior tube. **b** The flexure of the neural tube, as invoked by Wilhelm His (1831–1904). The flexure of the neural tube (right), is supposed to be analogous to the flexure of a rubber tube (left) (©Michael Holtz/Photo12)

### New forces driving embryogenesis: rays

However, vertebrates are more than just a set of nested tubes. At second order, they exhibit also a series of orifices, which, in simple vertebrates, such as the lamprey, present a striking geometry, as they are strictly aligned (Fig. 1a, left). The lamprey structure is so simple that it is even called the “flute fish”. In many fishes, the orifices are less round and have a slit shape. In terrestrial vertebrates such as humans the orifices have a more complex distribution (Fig. 1a, right). There is a general knowledge in classical embryology that the more complex distribution of orifices in humans along the anterior part of the body, arises from a forward flexure of the tubular pattern visible in the flute-fish (Fig. 1b, Ref. [16]).

We address in this article the physical mechanism of formation of these secondary structures, which form on the main shaft of the embryonic body. We point in particular to the distribution of forces able to generate such regular patterns. We have recently followed carefully in vivo the chicken morphogenesis [13], and we have found that the formation of bilateral orifices (ears, eyes, nostrils) and of the mouth arise from a second set of “purse-strings” which have an almost *radial* pattern in the early embryo, in addition to the known pattern of circular “purse-strings” [8, 9]. Hence, while it was first found that there are pulling *rings* in the embryo, now we put forward that there exist also *rays*, or pulling *radial* strings [13].

### Origin and effects of the bi-axial force pattern

**Fig. 2:**
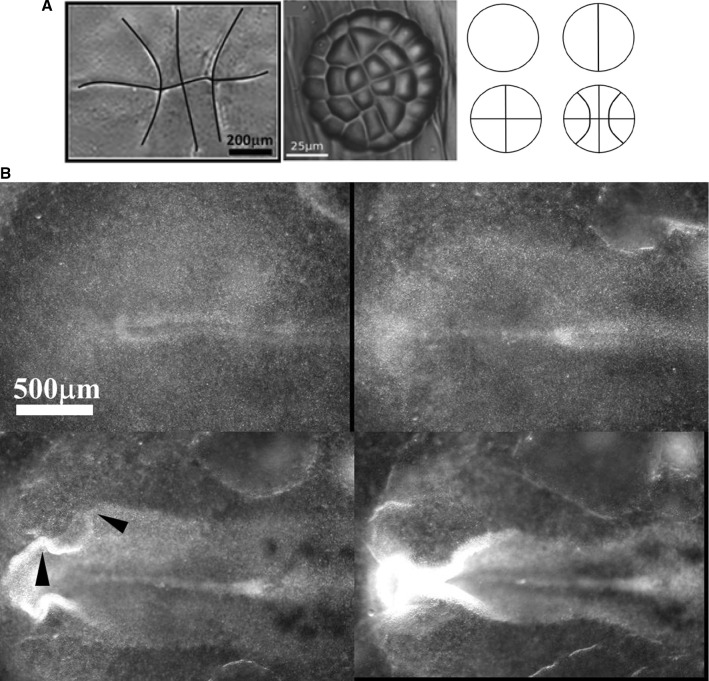
**a** Left: The first three rounds of division in a chicken generate a pattern of a cross with curved periclinal lines (tend to be radial peripherally, but reconnect at right angles in the center, photograph Richardson [19]). Middle: Pattern of division in the plant (*Dionaea Muscipula* [17]). Right: The sequence of cleavages. **b** During neurulation, the folds roll up to form the neural tube. The folds exhibit kinks (arrowheads) corresponding to the position of the future head features, especially the eyes. These kinks are locked to lines visible along the flat blastula, prior to neurulation. The images are taken from time-lapse chicken embryo observation under a microscope at mag. 4X (Video 1)

The set of rays or radial strings originates in the early pattern of cell cleavage [13], which is biaxial. Early zygote cleavages form two set of lines at right angles, because the equilibrium configurations of minimal cleaving planes tend to be at right angles, after cleavage [17, 18]. This equilibrium configuration is observed both in plants and animals [13]. It is remarkable that the three first rounds of cleavages are strictly identical in a plant and in the chicken embryo. Hence, there exist both circular and radial lines^1^ in the reference configuration existing prior to embryogenesis (Fig. 2).

In animals, the cell cleavages occur by constriction of a lasso of actin-myosin [20]. Therefore, the lines of cell cleavages are seeded *ab initio* with actin-myosin complexes. These lines are still able to constrict later during embryogenesis, albeit at a multicellular scale (during embryogenesis, cell cleavage splits existing cells into ever smaller cells, but the initial broken symmetry is conserved since it is part of the boundary condition for the cleavages).

**Fig. 3:**
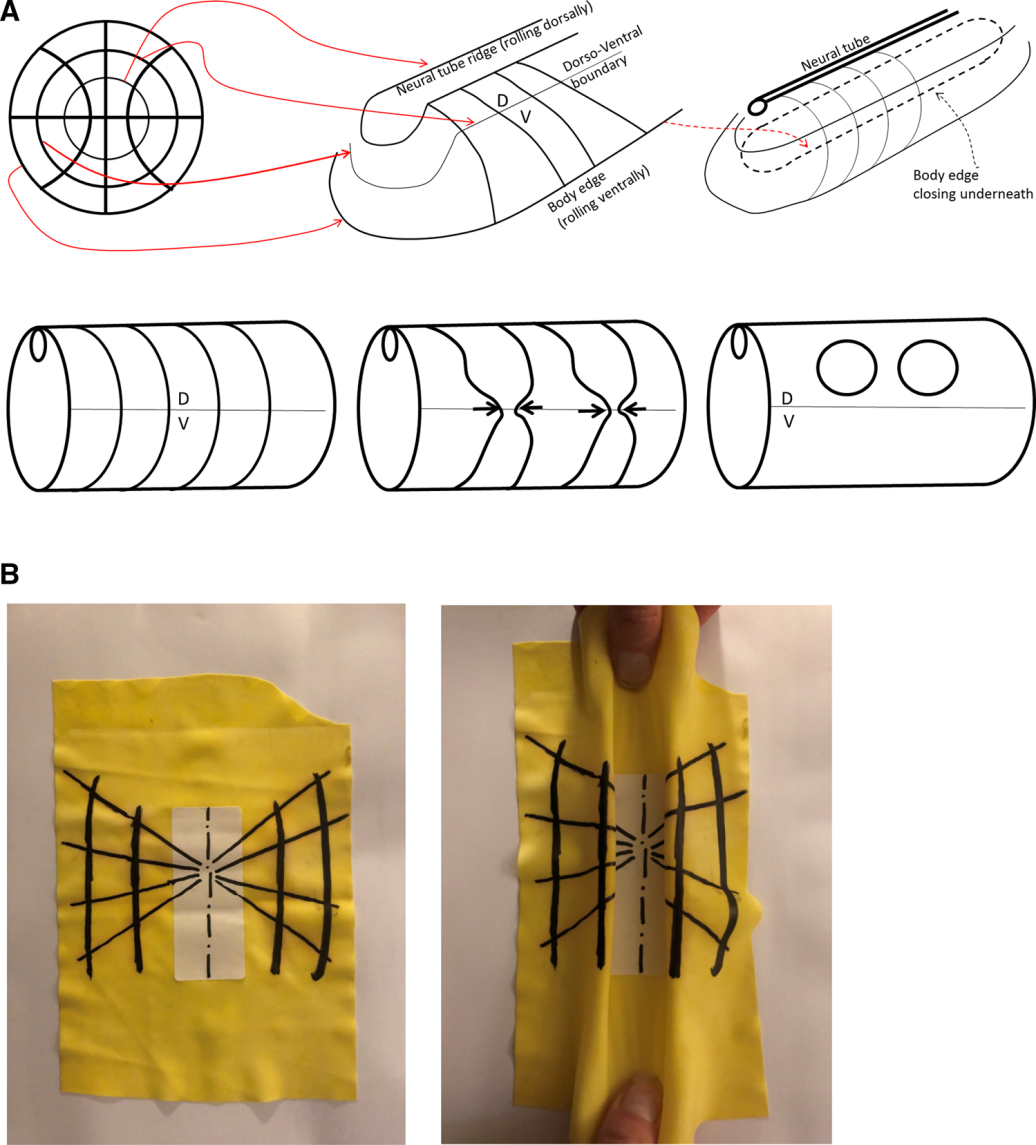
**a** The early pattern of cleavage of the chicken oocyte has lines with intersections at right angle (top Left). After many subsequent rounds of divisions, the embryo folds along a pattern of domains inherited from the first divisions (top middle and right). The concentric boundaries serve as template for “purse-string” contractions, which cause the formation of an Antero-Posterior tube. The central circle forms the neural tube by rolling up dorsally, one external circle forms the body boundary by rolling down ventrally. The radial lines find themselves forming lines on the shaft of the tube bottom left. The contraction of these lines generates the orifices along the shaft (bottom middle and right). **b** Toy model of how a crenel is generated during neurulation of a radial and orthoradial pattern of lines. We take a piece of rubber foil, and stick a tag to get a central stiffer area. We next pull on the rubber and form a buckling instability which tends to generate a tube (*in vivo*, the force has different origins, such as mesodermal pull [7], and cell intercalation [21]). The pattern of lines is dragged towards the median axis, and it rolls up so that crenel-like domains are formed along the “flanks”

During embryogenesis, the circular “purse-strings” contract first (by day 1 in a chicken) and they generate the Antero-Posterior (A-P) tubular form of the basic vertebrate *bauplan* [5, 7–10]. Figure 3 shows the roll up of the embryo body forming a tube, called “neurulation”. During embryo roll-up, the presence of these lines is already visible in the kinks evidenced along the edges of the folds (arrowheads in Fig. 3) due to the presence of the radial texture. At the end of neurulation, sensory organs are absent. The contraction of the radial lines (now thickenned) occurs a few hours later (by the end of day 2 in a chicken), while the first lines are still constricting. This generates lateral features on the said Antero-Posterior tubular form, especially, the ears, the eyes, the nose [13]. As these features are orifices diving into the brain, they naturally form sensory organs.

During tissue roll-up, the first pull by the circular lines deform slowly the lateral sides (called plates in embryology) of the entire blastodisc, such that the rays and sectors formed by the second set of strings are deformed and advected on top of the dorsal side of the embryo (as in Video of neurulation Video 1). The deformation projects the radial lines existing on the blastula on a crenel shape along the dorsal side of the embryo body, Fig. 3a and b. shows an all-elastic analog of the phenomenon : the longitudinal buckling (folds perpendicular to the medio-lateral axis) advects the radial pattern and projects it dorsally in a pattern of crenels. At this stage the actual vertebrate body forms roughly a cylindrical tube with such a crenel like structure in the ear area, and a more elongated structure (the eye stalk) in the eye area [13].

In the chicken embryo, a few hours after the formation of the body cylinder, the second set of strings contracts [13]. This contraction generates the mouth, and the sensory organs (the order of formation of these organs is Mouth, Ears, Eyes, Nose (see Video 2, showing that eye formation is posterior to ear formation, and Video 3 showing that nare formation is posterior to eye formation), by the mechanisms described here.

Therefore, the vertebrate plan, in its less derived form, arises from a biophysical process acting in two steps: minimal cleavage of a disc, followed by contraction along the lines of cleavage in the order: orthoradial and next radial, with force exerted in both cases by actin-myosin filaments [11, 14]. In addition, we have shown that the forces are excitable and that they trigger each other in cascade [10].

### The constitutive equation for the material

In general terms, when addressing a given developmental phenomenon, one needs to find, in addition to the pattern of forces, the constitutive equation for the material. This relates stresses to deformations or deformation rates. Living material is a complex gel with visco-elastic properties [20, 22], and crystal liquid-like orientational order [23]; moreover, biochemical activity may change the material properties during development [24]. However, at early stages embryonic tissue is poorly differentiated and quite soft [25]. For the sake of simplicity, we shall assume here, following a number of authors [22, 25], that the early embryonic material is a simple material, viscous in the long time scale, albeit with a bulk viscosity, in addition to shear viscosity. Also, we have shown previously with localized air puff tonometry that the early embryonic material exhibits a simple creeping flow under stress [26], and that gastrulation is a viscous vortical flow [5, 7]. Technically, the embryonic material will follow hereafter a constitutive equation of the form $[\upsigma ]=[\mathbf{H} ][\partial _{t} \upvarepsilon ]$ where $[ \upsigma ]$ is the stress tensor, [**H**] the viscosity matrix, formally analogous to a Hooke tensor, in which the components are identified with viscosities instead of elastic parameters and $[\partial _{t} \upvarepsilon ]$ the strain rate tensor. The displacement rates are obtained quasi-statically, by solving numerically the equation $\mathbf{div} [ \upsigma ]=\mathbf{0}$ in the bulk with given forces at the boundaries. The deformed configuration is updated incrementally and used to calculate the new displacement rate field in a Lagrangian specification. A similar *ansatz* has been proposed for numerical simulation of drosophila development [27]. In a previous work [9], we have shown that the total force exerted by one of the embryonic rings is of order $10^{-6} \,\hbox {N}$, and that the local deformation rate at cell level is of the order of $10^{-3}\, \hbox {S}^{-1}$, with a total width of the body (say in the neck area) of the order of $500\upmu$m; the elasticity of the embryo is in the 1000 Pa range. In the simulation we take elastic parameters and dimensions giving a similar material characteristics (NB: in the numerical simulation the actual value of $\hbox {E}=2$, but over a dimension of 0.4, which, when dimensioned by the actual size gives a physical value of E in the proper range).

### Modeling of the bi-axial force pattern

As we are interested in formation of the sensory organs, especially the ear, our reference configuration will be the embryonic body by the end of neurulation, when the disc has rolled-up to form a tube, especially in the area of the presumptive ear. In Part II hereafter we shall invoke a simple lateral projection, or profile view, of the body, in order to model movements along the body as if a flattened tube. Invagination of the sensory organs occurs by inwards surface flexure, with the organ tissue diving down under contraction force (on a convex hull, contraction produces a Laplace resulting force oriented inwardly). The sensory organs are therefore, schematically, deformed crenels of nematoid cells, forming cylindrical tubes by diving down inside the head. In this article we shall not address the inwards flexure of the tissue but only the surface movement which positions the sensory organs domains. We shall only report a 2D viscous calculation, showing that, by varying empirically the values of the tension gradients along the orthogonal lines, we can reproduce qualitatively several features of existing animals. However, the mouth invagination, as seen in profile view will be addressed.

The model makes one specific prediction : the contraction slows down, and the formation of the animal, in the absence of growth, is actually a self-arresting process. However, actual nutrient uptake contributes to growth, especially in birds and mammals, which either feed on the egg or on the mother, during development. Growth is most often regulated by hormones up to puberty. However, many animal species such as jellyfish or crustaceans may grow indefinitely in a growth mode called indeterminate growth [28]. Still, growth is less a morphogenetic process than development, and, sensibly, medical science distinguishes development and growth^2^ in that growth is characterized by a change in scale due to nutrient uptake and growth hormones, but no true development. (For example, the hand may grow from a size of approx. 1 mm to meters long as in whales, without formation of new fingers). Contraction forces reshuffle and reshape the tissue by quadrupolar shear flows of tissue which are conservative, this is why most embryos, such as fish, may actually form without growth (by quadrupolar we mean a conservative flow with a saddle-point generated by two forces oriented head on. A single force is a dipole of pressure in a simple potential flow hypothesis). Even in birds and reptiles, yolk uptake occurs after the heart is formed and the vascular loop is established, which is by day 4 of development [29]. In the view presented here, with forces slowly shrinking during morphogenesis, embryo formation is a form of wound healing under contraction, prior to growth. This is why we start by describing that phenomenon in part II.

**Fig. 4:**
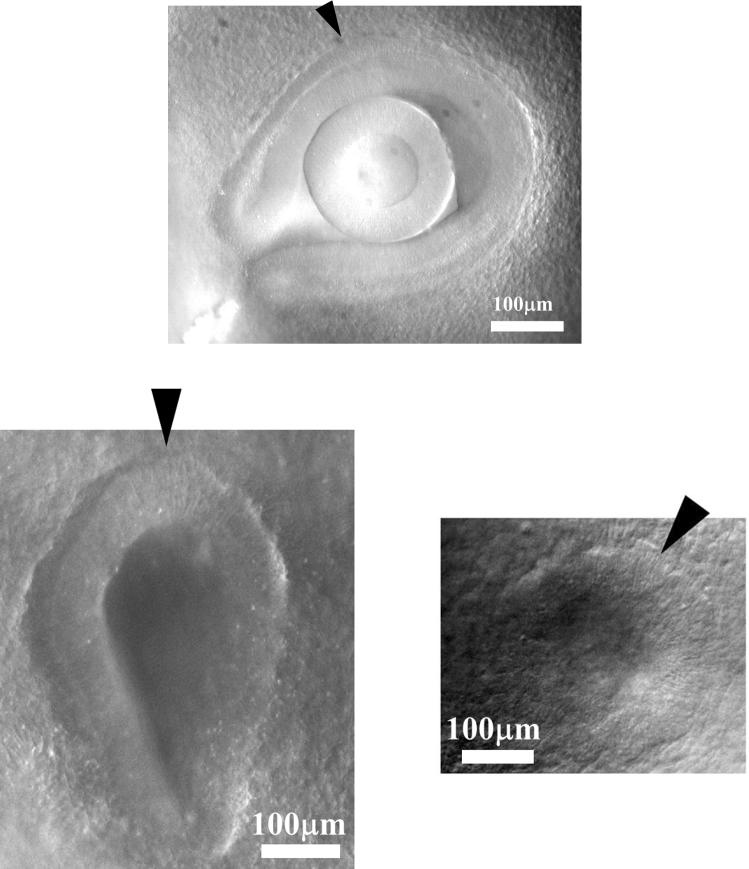
As sensory organs form, cells progressively align along the ridge of the organ (arrowheads). Top, a chicken embryo eye by the middle of day 3 of development, bottom left an ear by the end of day 2, bottom right a nasal pit by the end of day 3. The stacking of cells organizes itself in a dorsal to ventral progression see Video 7). This stacking of cells favors anisotropic growth of the tissue. The ridge behaves as a growing line

While we shall not include bulk dilation forces in the model, in parts II.5 and II.6 we shall however include growth along the boundaries of the texture (radial and orthoradial lines). Indeed, the forces along the strings are not always contractile. Actually, the cells will progressively align or stack parallel in a conspicuous orientational order (Fig. 4), along what is called a “ridge” or “crest”. The early stages of nasal pit, ear pit and eye ball, exhibit such a nematoid stacking with parallel cells following a defined contour (Fig. 4). This alignment transforms progressively the “purse-string” into a cellular ridge [19] which grows anisotropically. The anisotropic growth of such a ridge or crest produces an extensional work, which behaves as a negative linear tension giving rise to a development oriented outwardly. The cells in the ridges stack in a spatio-temporal order oriented away from the median axis, i.e., they start stacking at the point of contact with the neural tube, and progress until reaching the ventral side. We assume that contact with the neural tube favors cellular tension as observed in Ref. [22]. This will also justify the introduction of a tension gradient in part II.2.

## Results

### Closure of a ring in wound healing

The simplest mechanism of wound healing consists of a circular edge which constricts like an iris shutter until the hole vanishes (the topological singularity of the hole is resolved). This situation is the one which is observed in experiments of tissue puncture, such as puncture of the amniotic sac [6]. Now, this experiment is actually identical to the physiological situation by which the amniotic sac itself forms and closes [9, 30].

We can simulate this situation by considering a uniform tension along an edge. We define a domain with a boundary (a circular hole) and apply along the boundary a tension **T**, along the tangent vector **t**. The tension along the contour results in a surface force acting on an element of contour which is $\partial \hbox {T}{} \mathbf{t} /\partial \hbox {s}$. There are classically two terms, the Laplace centripetal force representing the effect of a constant tension **T** along a curved contour : $\hbox {T} \partial \mathbf{t} /\partial \hbox {s} = \hbox {T/R}\, \mathbf{n}$, and the tangent force related to the variation in magnitude of $\mathbf{T} : t \partial \hbox {T}/\partial \hbox {s}$ we assume in this first calculation that the magnitude of the tension T is a biochemical constant. In this case, the Laplace term is akin to a pressure.

**Fig. 5:**
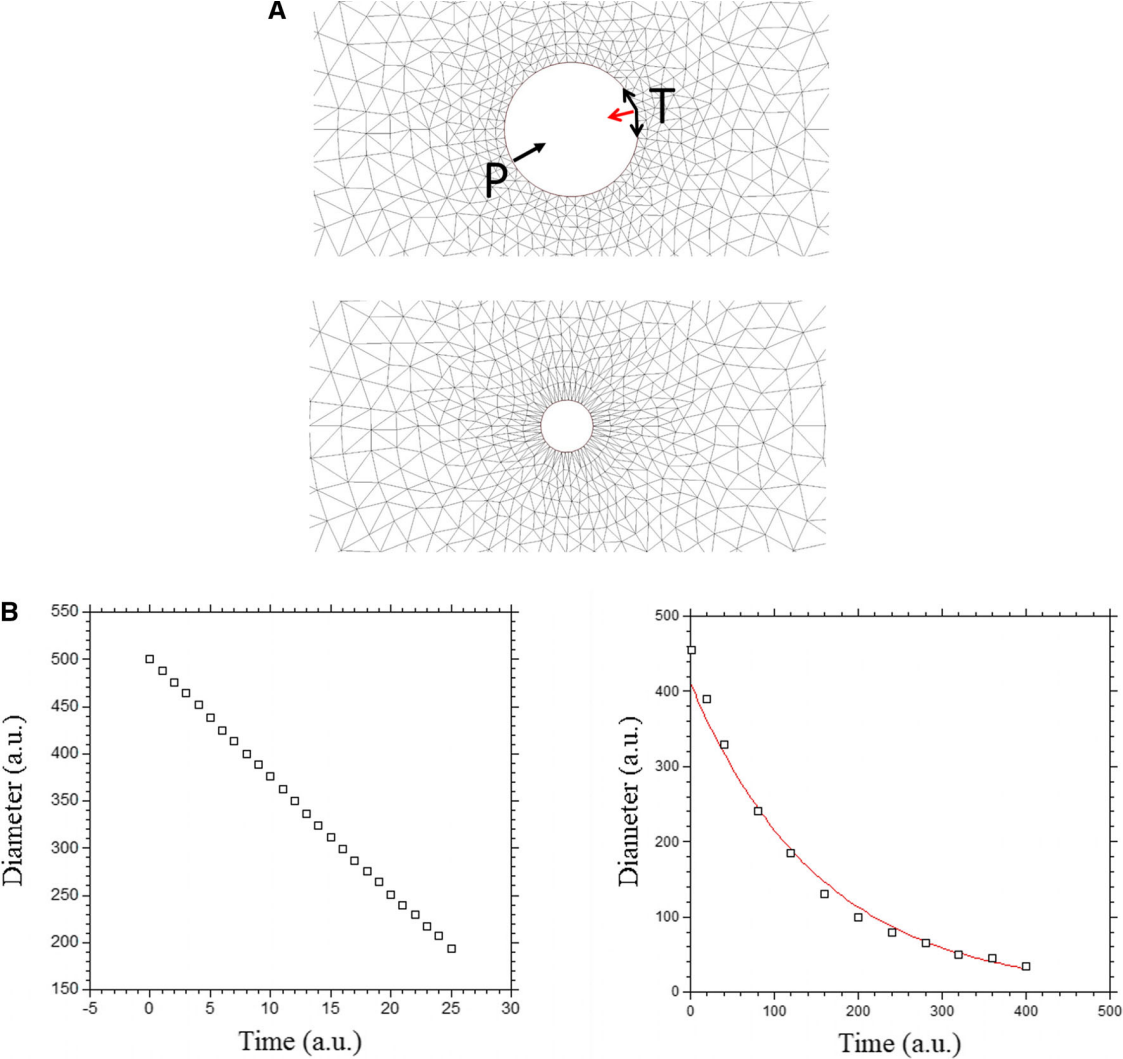
**a** Numerical simulation of closure of a circular wound at constant tension, or at a constant pressure. At constant tension, the curvature of the contour implies a radial equivalent Laplace pressure (red arrow) with value $\hbox {T}\upkappa$, with $\upkappa$ the curvature. The Laplace pressure increases with time, while the perimeter decreases, so the total force is constant. At constant pressure P, the pressure is constant, and the radius decreases, so the total force decreases. **b** Left, shows the plot for a constant tension: the healing is linear in time (Video 4). **b** Right, shows the numerical simulation of closure of a wound at constant pressure: it shows an exponentially slowing down healing (the proposed fit is to an exponential)

We solve for the displacement rate with our viscous hypothesis, by finite elements (FE) methods. We assume an explicit discretization in time. We use the solver Freefem$++$ for this calculation, and the MoveMesh macro to displace and update the grid incrementally after each calculation [31]. Each deformed, updated, configuration becomes the reference configuration for calculation of the new deformation rate, for the next step. The edges are labeled and the force term is kept constant along the internal hole. The curvature at the hole boundary is calculated using the curvature routine proposed by Dapogny et al. [32]. A normal force term akin to a pressure is generated at the boundary whose magnitude is $\hbox {T}\upkappa$, where $\upkappa$ is the curvature. We assume here that the tissue itself does not produce an expansion or growth pressure.

We find that as the diameter of the hole decreases, the perimeter of the pulling edge decreases (Fig. 5a), so that the hole tends to “ heal ” with a linear slowing down (Fig. 5b, left, video 4). Such ring closure is observed, and it indeed proceeds almost linearly during embryogenesis (Fig. 5c, video 5, see also Ref. [30]). Now, we see that as the perimeter decreases, the curvature increases, so that the equivalent centripetal pressure due to tension increases, as the diameter decreases. So although the pulling length (the perimeter) is smaller, the total force (the integral of the centripetal pressure along the perimeter) remains constant. This is the cause of the linear decay, in this geometry. If instead of considering a constant tension (which implies an increasing centripetal pressure during healing), we assume directly a constant centripetal pressure, we get instead an exponentially slowing down closure of the hole, since now the total force decays (Fig. 5b right).

**Fig. 6:**
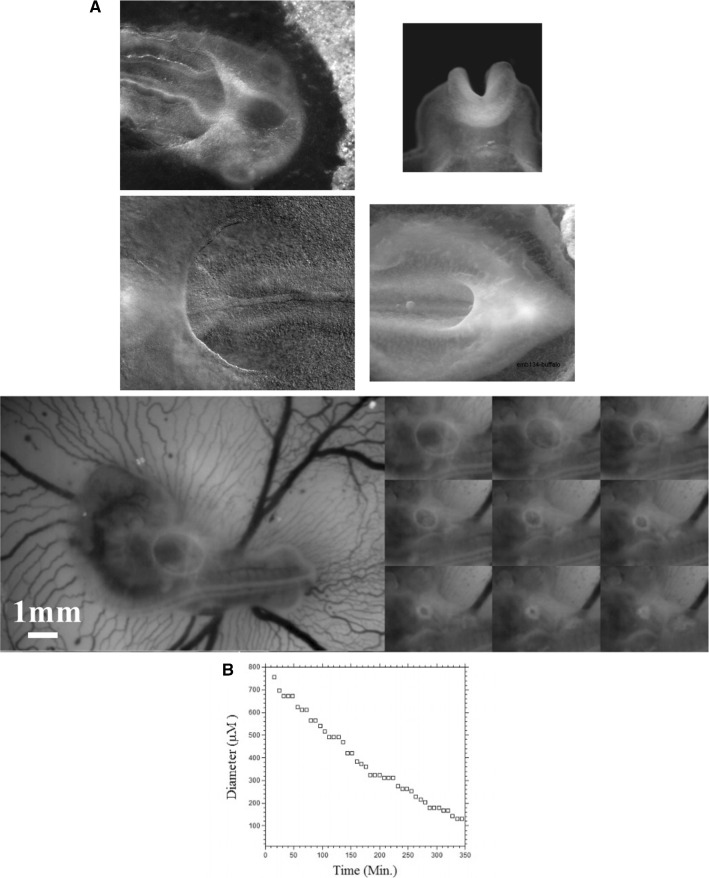
**a** Contracting rings are found during formation of the amniotic sac (top left, anterior photo of an early day 3 chicken embryo in dorsal view) of the heart (bottom Left, anterior photo of a late day 2 chicken embryo in ventral view) of the head (top right, closure of the neural tube, photo of an early day 2 chicken embryo in frontal view), during formation of the hindgut (bottom right, posterior photo of a late day 3 chicken embryo in ventral view). **b** In vivo time-lapse video-microscopy of amniotic sac closure in chicken embryos (video 5). To the right, the temporal evolution of the hole diameter, showing a close to linear closure of the hole

In amniotic sac formation, a ring of cells forming a purse-string constricts up to the point that the amniotic sac is closed. A similar phenomenon is observed in heart formation, in head formation, or in ventral closure (Fig. 6a) (and also in early stages of limb formation [10]). The closure of the amniotic ring is linear (Fig. 6b), and so is the closure of the heart ring [30]. This is the basic principle by which embryogenesis occurs : cellular myogenic forces are actually contractions, and the contractions tend to shrink the source term self-consistently, thus leading to self-arrest. This shows that purse-string contraction is central to animal formation, in addition to be a mechanism of wound healing and regeneration [6, 9].

### Free linear contraction and ear formation

**Fig. 7:**
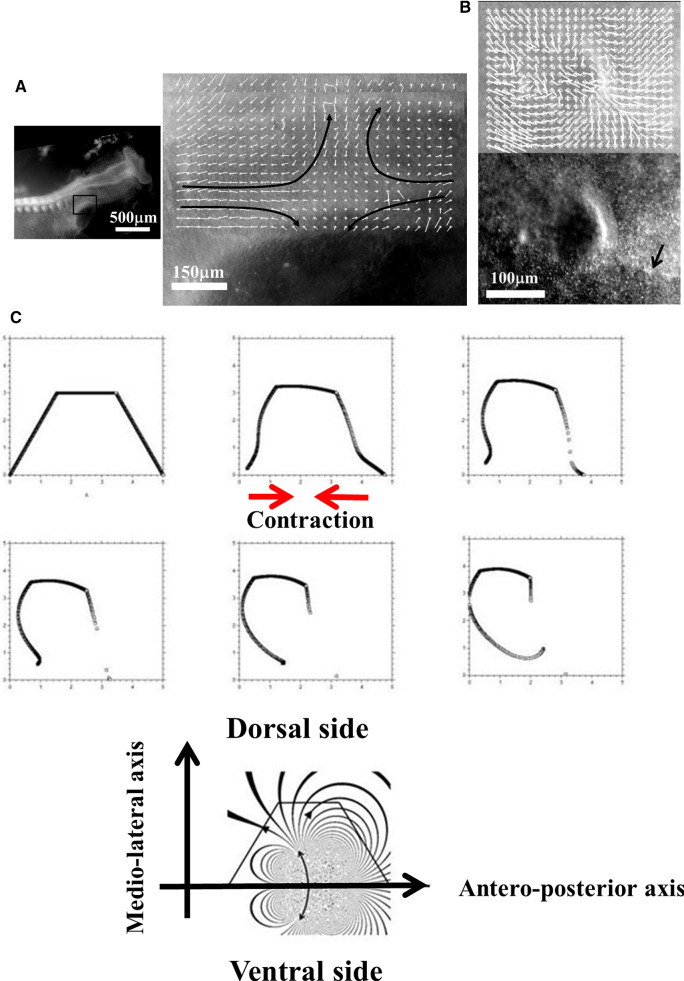
**a** (Adapted from Ref. [13]. Prior to ear formation the area of the presumptive ear undergoes a contraction along the dorso-ventral boundary, centered on the future ear movements (extracted by Particle Imaging Velocimetry on time lapse movies). **b** A similar contraction along the dorso-ventral boundary (arrow) is observed in the eye territory prior to eye formation, which is best visible at the earliest stage during neurulation. The in plane “eye placode” undergoes a contraction, and an inwards winding which can be extracted by Particle Imaging Velocimetry. **c** Modeling of the asymmetrical winding of a trapeze contracted along its base gives ear-like patterns (Adapted from Ref. [13])

We now turn to more complex geometrical situations as found along the body of vertebrates. We have shown recently that ear deformation occurs by contraction of the dorso-ventral boundary (Fig. 7a). For this present article, we have also followed early stages of eye formation, and we also evidence a deformation by contraction along the dorso-ventral boundary (Fig. 7b). We have proposed in Ref. [13] a simple model of potential flow, able to reproduce ear formation by rounding off of a sector of tissue which constricts, under the effect of a contraction occurring along a line (the Dorso-Ventral boundary D-V). In this model (Fig. 7c), a sector of tissue is advected in the flow induced by the contraction of the base of the trapeze. The flow is supposed to be 2D, and the fluid speed is obtained by the potential vector and stream function technique, with $\mathbf{V} (x,y,0)= \mathbf{curl} (\mathbf{A} )$ and $\mathbf{A} =(0,0,\Psi (x,y))$. We solve for the flow generated by forces represented by two vertical lines of cells located at $\hbox {x}=\hbox {a}$ and $\hbox {x}=\hbox {c}$, with a pull exerted by cells in the vertical segment along $[-b,b]$ in Oy. The pull by the two sources has opposite signs along Ox, so that the forces are oriented head-on in a contracting manner, and they have different magnitudes to provide an asymmetry describing head flexure (the magnitudes are represented by the parameters $\upalpha$ and $\upbeta$ in the formula below). They are located in the drawing Fig. 7c to the right and to the left of the trapeze. This leads to solving the equation:

1 $$\begin{aligned} \Delta (\Psi (\hbox {x,y}))=\hbox {F}(\hbox {a})+\hbox {F}(\hbox {c}) \end{aligned}$$

The function $\Psi (x,y)$ is found to have the analytic expression:

2 $$\begin{aligned} \Psi (\hbox {x,y})= & {} \upalpha \hbox {ln}((\hbox {x-a})^{2}+(\hbox {y-b})^{2})-\upalpha \hbox {ln} ((\hbox {x-a})^{2}+(\hbox {y}+\hbox {b})^{2})\nonumber \\&-\upbeta \hbox {ln} ((\hbox {x-c})^{2}+(\hbox {y-b})^{2}) + \upbeta \hbox {ln} ((\hbox {x-c})^{2}+(\hbox {y}+\hbox {b})^{2})\nonumber \\ \end{aligned}$$

The streamlines are the Iso-$\Psi$ lines shown in Fig. 7c. This flow advects the trapeze as in Fig. 7c. However, in this calculation, the force term is not advected in the flow, the contour flows in a constant flow. For the solution to be self-consistent, the force term must be modified as the contour changes, especially if the contour shrinks. Although the preliminary model presented in Ref. [13] is not self-consistent, it nevertheless gives a remarkably realistic ear shape (Fig. 7c), probably because at first order the broken symmetry of the pattern comes from the asymmetry of the quadrupolar contraction, and omitting to take into account the shrinkage of the source amounts to an artificial acceleration of the process.

**Fig. 8:**
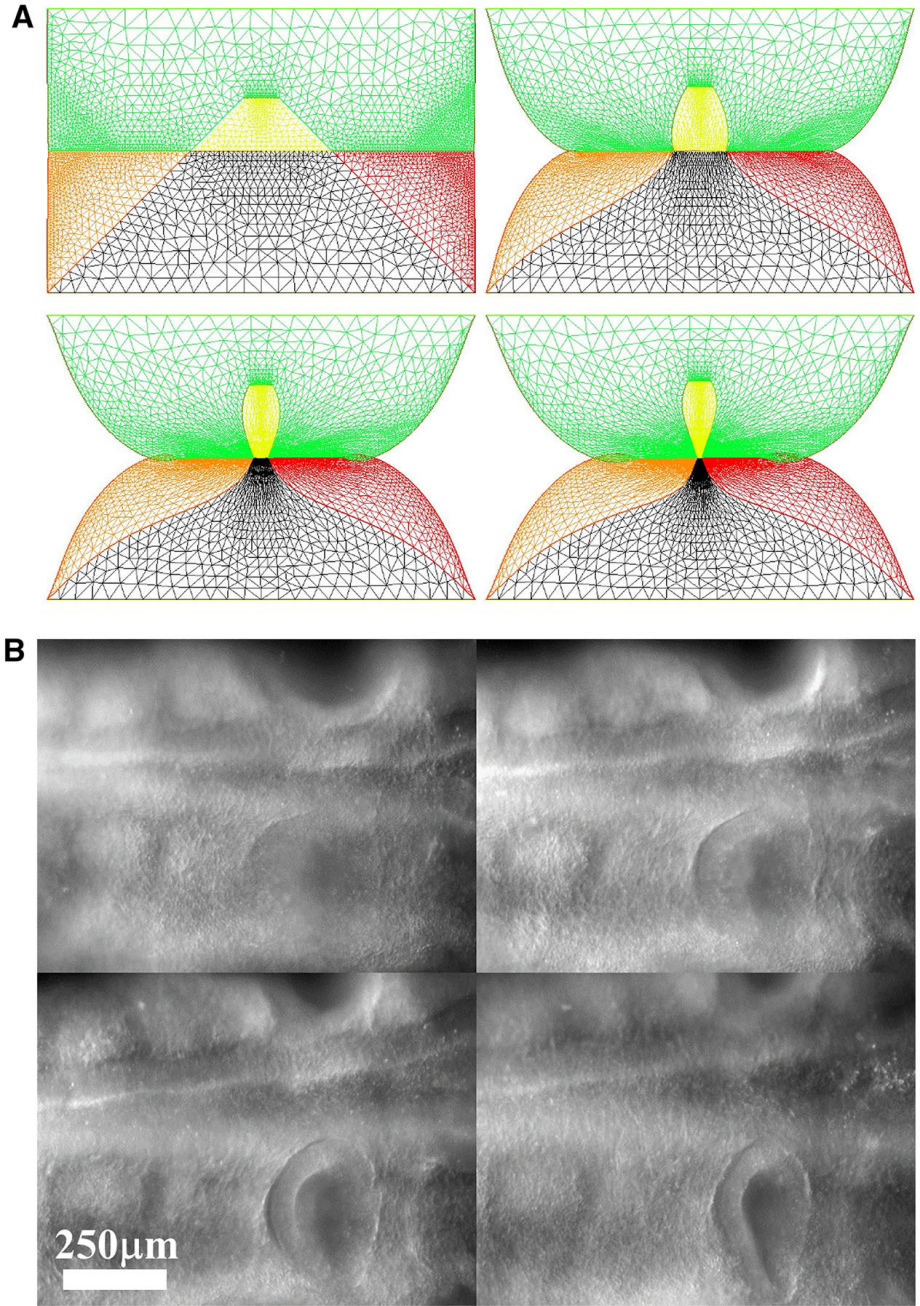
**a** Temporal evolution of a crenel during contraction along the D-V line. The crenel tends to round off, with a pear like shape having the narrow end oriented ventrally (Video 6). **b** In vivo time-lapse video-microscopy of ear formation in the chicken embryo. The initial crenel like domain is deformed and takes a pear like shape with the narrow end oriented ventrally (Video 7). One observes a progression of morphogenesis from the median axis, down. The morphogenesis correlates also with a progression in cell stacking in the presumptive ear ridge. **c** Slowing down of the morphogenesis in the numerical simulation as the contracting segment slows down. Following the initial deformation, the dynamics becomes close to an exponential slowing down (to the right the log-linear plot), linked to the fact that the smaller the segment, the smaller the force. **d** Effect of a gradient of tension in the ear edge (Video 8). During embryo development, there exists a media-lateral bias : the ear starts to form close to the neural tube, and patterning progresses from the neural tube downwards. Surface movement, buckling and cell alignment progresses in the same direction (see Fig. 8b). We assume the existence of a tensile gradient oriented from the median axis and outwardly away laterally with a maximum at the median axis. We introduce such a tension gradient as a surface shear oriented tangentially in the direction of the neural tube. This gives rounder ears. The pattern corresponds also to the deformation of the eye territory observed in Fig. 7b. **e** Effect of tension asymmetry (Video 9). During embryo development, there exists an A-P asymmetry of tension forces due to the heart pull along the ventral side. We assume an asymmetry between the tension along the DV line, between anterior and posterior, and also an asymmetry in the crenel tension, and follow the evolution of the ear form, the boundary conditions being fixed. We find a biased ear pattern reminiscent of ear form in vertebrates. **f** Comparison of an embryonic ear at day 3 of development (chicken embryo) and of a simulated ear. The arrow points to the vestige of the crenel which is advected towards the top in the simulation, and in the in vivo time-lapse movie. **g** If we assume a very soft, or even absent tissue in the trapeze, we observe the formation of a slit, instead of the formation of an ear (Video 10). This may explain the lateral collapse of the ear at the moment of inward buckling in 8B bottom right


We wish here to go one step forward and advect the force term itself in the flow. For this, we start from a 2D situation which is inspired by the situation in the embryo at a stage corresponding to end of neurulation, when the embryo is tubular. At this stage flanks roll-up has dragged the lateral tissue perpendicularly to the cylinder axis. Previous blastula sectors now form crenel-like trapezes, or hairpins of tissue intersected by the D-V boundary. In profile projection, our calculations will have the mouth to the left and the neck to the right. The large rectangle in Fig. 8. A is supposed to be a lateral projection of the body. The colored domains can be viewed as a representation of the biological mosaic inherited from early cleavage sequence. They correspond to switches in differentiation pathways induced during the first cleavages.

Now, we assume a slow contraction of the D-V boundary (Video 6). This situation is different from the one around the hole in that the edge along which contraction occurs is straight. In this situation, there is no curvature and solely the gradient of **T** matters. We assume a stress gradient contracting the ear. The linear gradient of stress dT/ds gives a line-force **F** tangent to the line. We assume it to be constant. We implement the line-force contracting the ear domain along its bottom boundary in Freefem, by introducing a constant shearing term along one half of the pulling segment, and the opposite value along the other half. Thus both forces, equal in magnitude, are oriented towards the ear territory, and they act antagonistically along the base of the crenel or trapeze. This creates an inwards winding of the ear domain.

We observe a progressive rounding off of the crenel, as the entire domain constricts, and the formation of a pear-like ear (Fig. 8a, Video 6), actually quite similar to the early stage of embryonic ear (Fig. 8b, Video 7). Since the segment shrinks, the movement slows down progressively (Fig. 8c), with a close to exponentially slow arrest, similar to the one discussed above. This is not surprising since a linear viscous flow problem with a linear source, will see a flux at the tip of the line proportional to the magnitude of the sources inside the domain comprising the entire line, so the flux will be proportional to the line length; therefore, the shortening speed of the segment will behave as:

3 $$\begin{aligned} \hbox {dL}/\hbox {dt} \propto -\hbox {L, hence L} \propto \hbox {exp}(-\hbox {t}/\uptau ) \end{aligned}$$

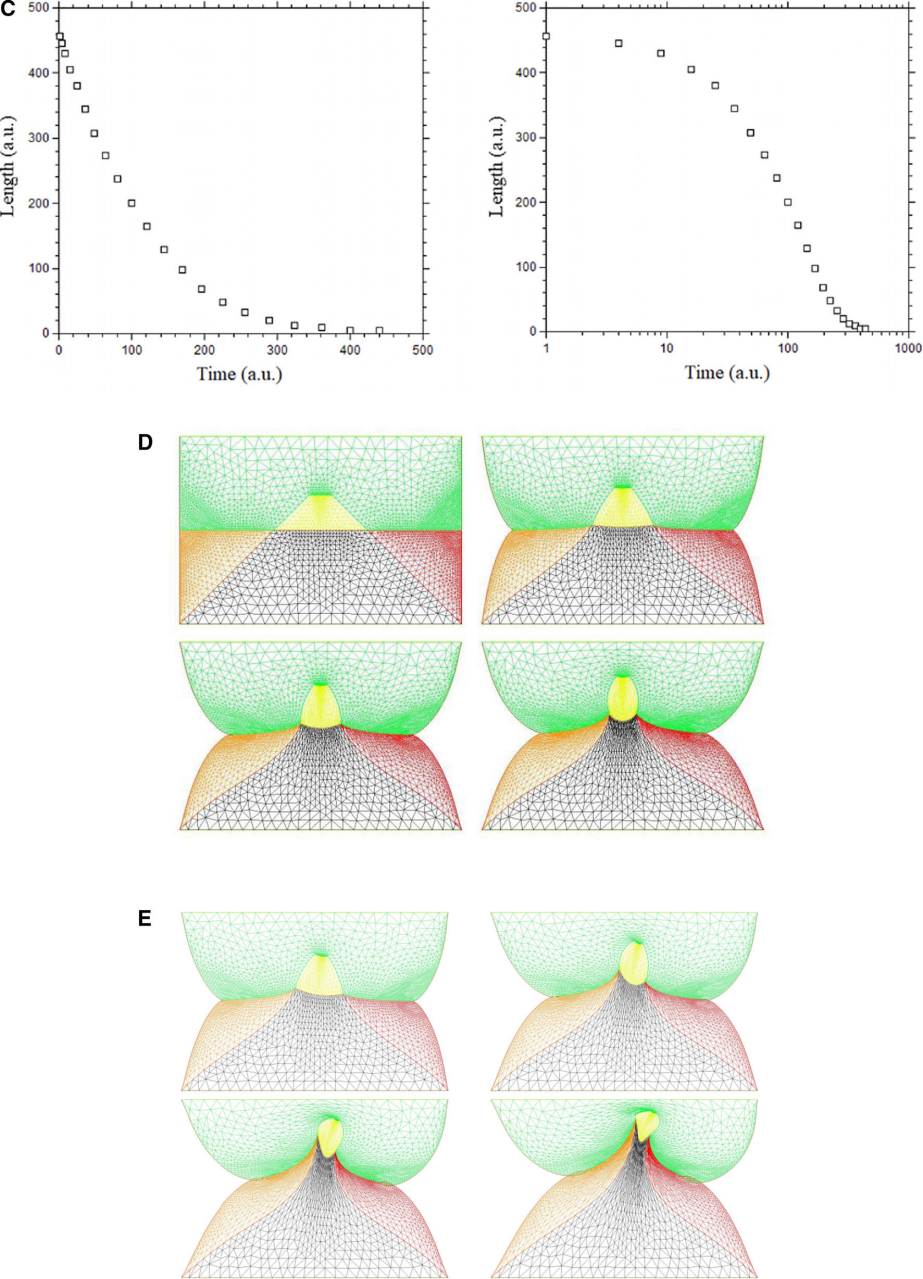

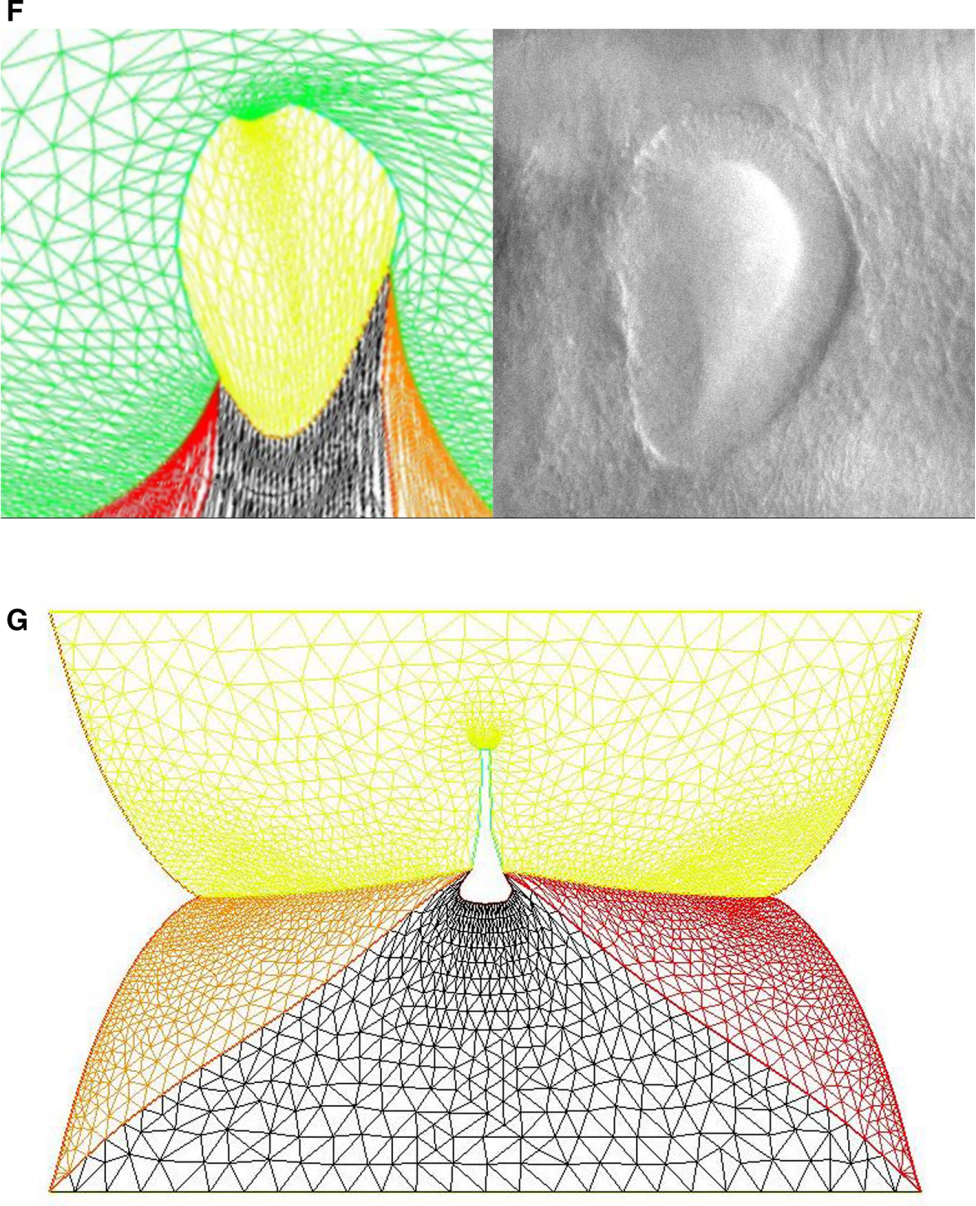


Now in this calculation, the shape of the ear tends to form an elongated horseshoe. We can observe (Fig. 8b) that in the actual ear, both tissue buckling and cell orientation propagate from the median axis (dorsal Antero-Posterior axis of symmetry), towards the ventral side. We ascribe this effect to a gradient of tension from the neural tube out. We can introduce the effect of a gradient of tension by a tangential shear force oriented towards the neural tube. This produces a rounding off of the ear, and an advection of the trapeze towards the median axis (Fig. 8c, Video 8). Now, we can introduce also an asymmetry of the contraction, and start to produce asymmetrical ear shapes (Fig. 8d, Video 9), quite similar to actual embryonic ears (Fig. 8e). We also see that the trapeze is deformed in a way much similar to the deformed trapeze or hairpin that remains visible around the ears, even in adult animals.

We also addressed the situation when there is an actual hole, or extremely soft material in the ear territory, and found that, instead of a rounding off, the ear domain takes the shape of a slit (Fig. 8f, Video 10). This may describe qualitatively the formation of slits instead of ears.

### Mouth formation

**Fig. 9:**
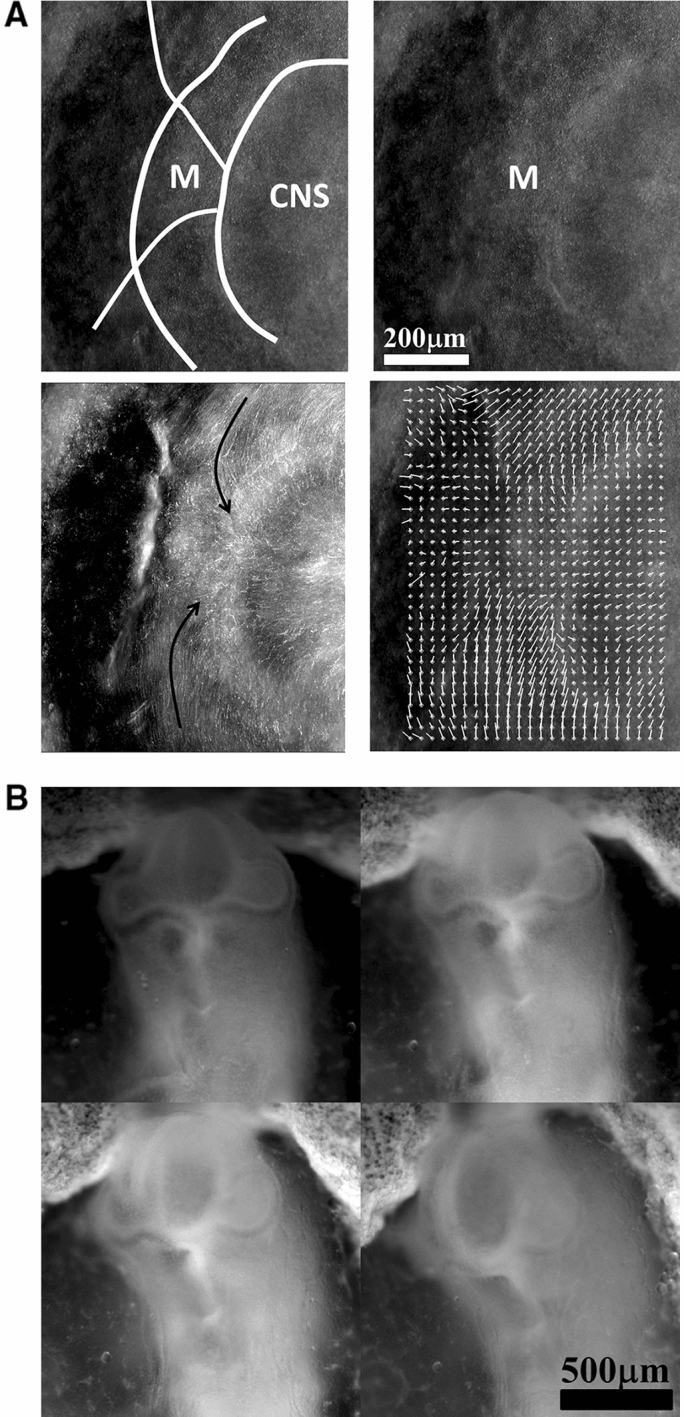
**a** In plane embryogenetic movement forming the mouth. Anterior to the left, posterior to the right. Initially the blastodisc is approximately round with an anterior sector already present (partly visible here by its darker contrast). The top left image shows the description in terms of radial and orthoradial lines, and the situation of the presumptive mouth. This anterior sector undergoes a contraction, quite similar to avian gastrulation, by which the sector constricts orthoradially. The bottom plates show the mouth area filmed here on the chicken blastodisc, in time-lapse video-microscopy (Mag. 10X, Video 11)). The bottom left image shows a simple overlap of the plates in Video 5, revealing the actual trajectories of the cells. The bottom right image shows the PIV (Particle Imaging Velocimetry, using NIH software by Wayne Rasband, and Tracker Plugin by Olivier Cardoso and Bérengère Abou) extraction, showing the vector field, with a pattern of contraction oriented towards the mouth sector. So during this phase, the contraction is mainly orthoradial (it follows the ring purse-string). **b** Continuation of mouth formation when the embryo is no longer 2D. As the embryo flexes, the mouth continues to form by a contraction of the edges which were previously the in-plane boundaries of a sector (Mag. 4X, Video $5=12$). As these edges constrict, the presumptive gums start to bulge out. **c** Pattern generated with an “ear generating contraction”, and a “mouth generating contraction”. The mouth area bends inwardly and two gums form, meanwhile the ear territory constricts and the ear crenel forms a hair-pin (Video 13)


We now turn to the foremost part of the “ tube ”, which is represented by the left vertical boundary in our simplified model. The mouth territory arises from a sector which constricts visco-elastically (Fig. 9a, Video 11). The contraction is first in-plane, and it continues out of plane, as it forms the gums of the mouth (Fig. 9b, Video 12). We implement a contraction of the mouth again by two symmetrical shearing forces oriented along the contour. We see that the mouth progressively invaginates (Fig. 9c, Video 13), in a pattern reminiscent to what is observed in vivo. In Fig. 8c, we have implemented both mouth contraction and ear contraction. Ear contraction pulls on the mouth area and tends to contribute also to mouth invagination. While we do not incorporate the normal component of the tension, it should be noted that the normal component would tend to hinder the invagination of the mouth as it works to straighten the boundary. However, for a segment flexing under a gradient of the form $\hbox {T}\approx \hbox {y/L}$ (T is maximum in the center, and zero by the edge, y is the vertical component along the boundary), the shearing force is $\hbox {F}_{\mathrm{xy}}\approx 1/\hbox {L}$ , and the normal term is of order $\hbox {F}_{\mathrm{xy}}\approx \hbox {x}/\hbox {L}^{2}$; therefore, it is initially negligible.

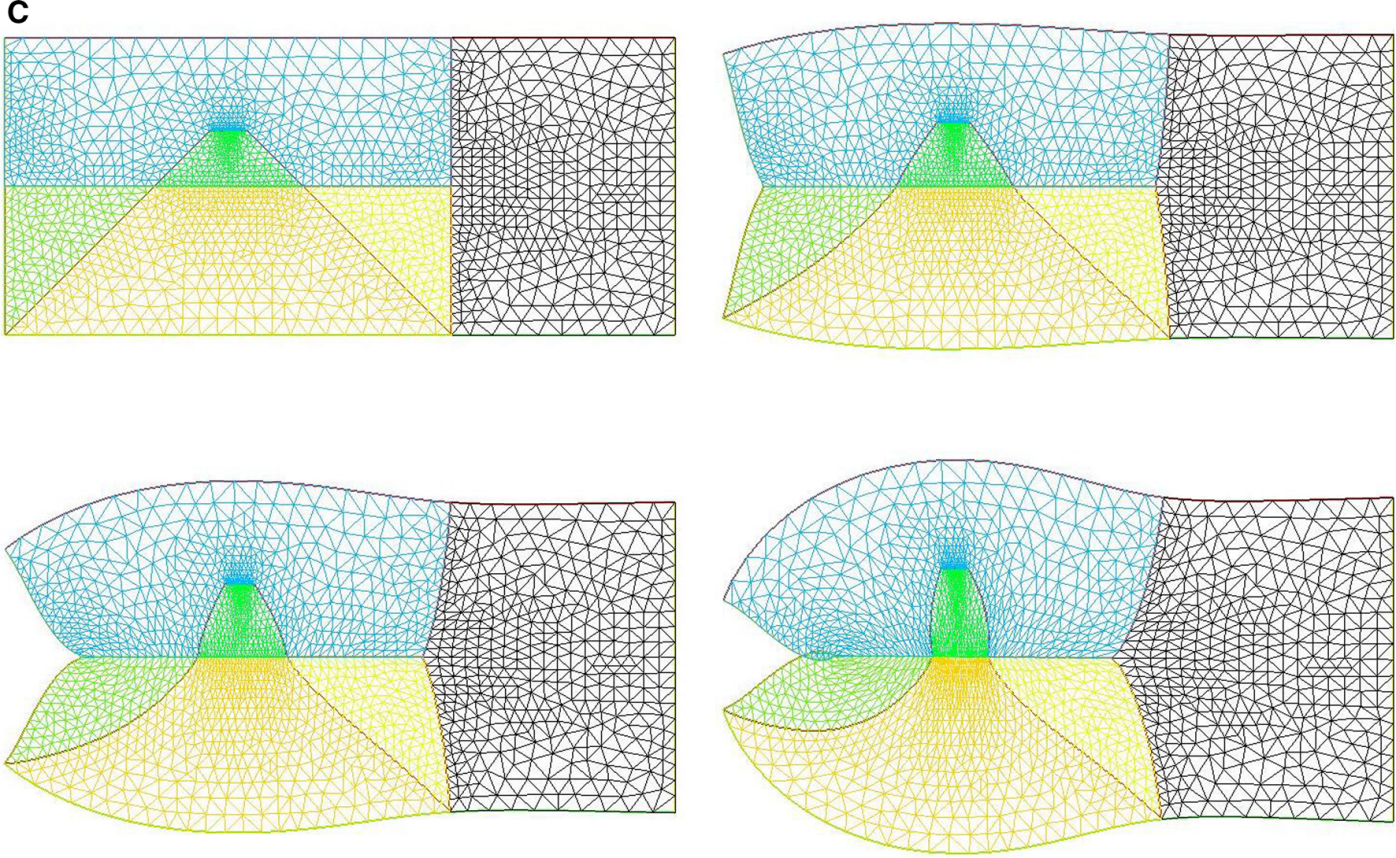


### Neck flexure

**Fig. 10:**
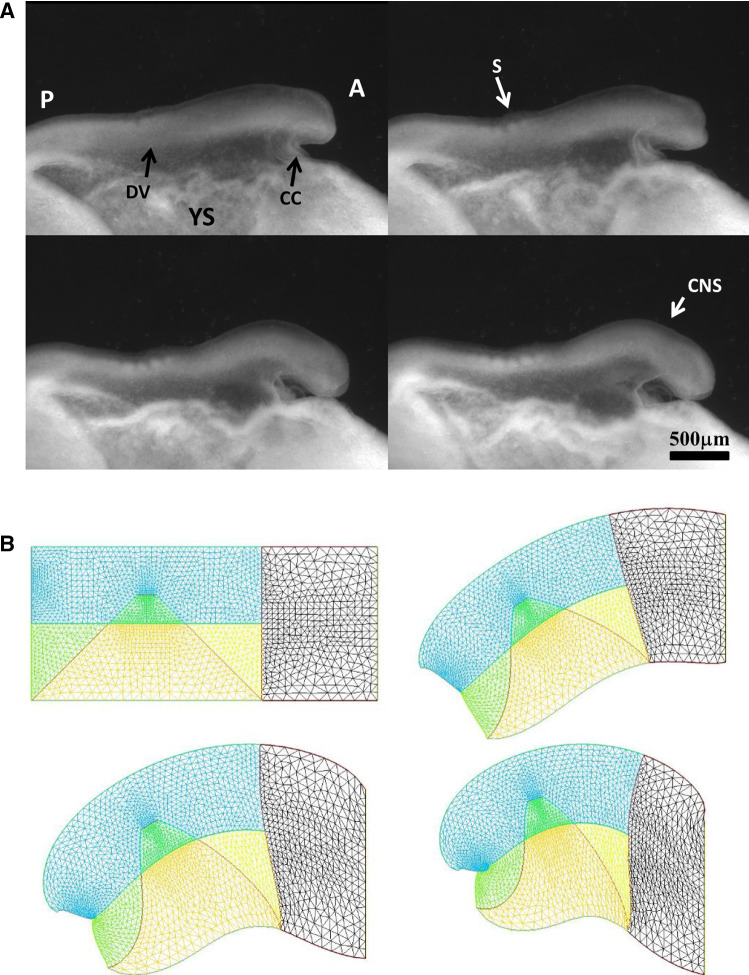
**a** In vivo time-lapse imaging of chicken flexure during the ventral pull, showing how the head part flexes ventrally (from Video 15). The movie shows the contraction of the cardiac crescent, and the forward flexure of the head. $\hbox {DV}=\hbox {Dorso-Ventral}$ boundary; YS$=$Yolk Sac; A$=$Anterior; P$=$Posterior; CNS$=$Central Nervous System; S$=$Somites; CC$=$Cardiac Crescent **b** Example of pattern built with a “mouth generating” contraction, an “ear generating” contraction and a “ventral” contraction (Video 16)

**Fig. 11:**
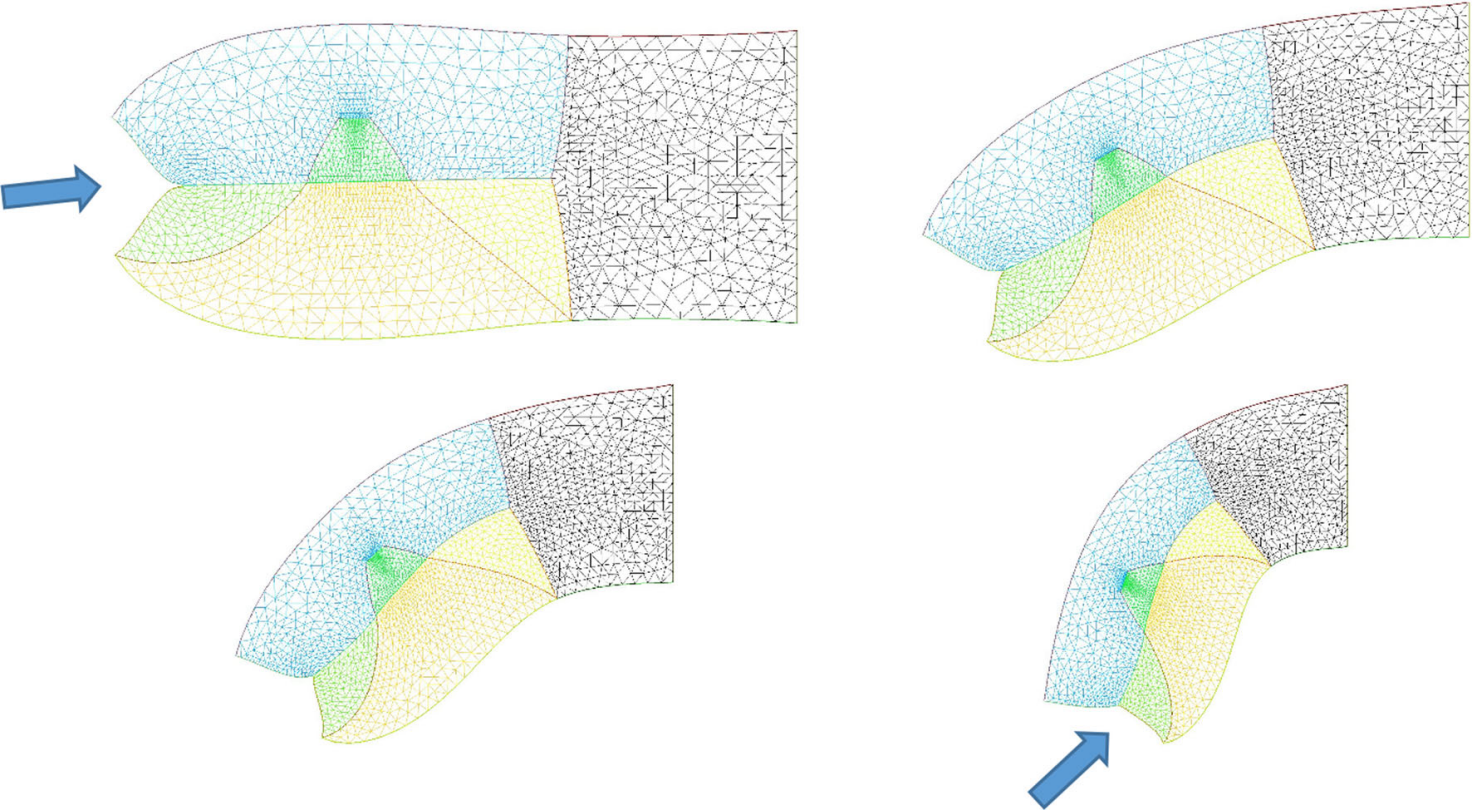
Neck bending obtained with a contraction of the ventral boundary. We start here from a situation in which the mouth invagination has started (top left). We observe that the neck flexure actually hinders the invagination of the mouth (bottom right, the mouth invagination is less pronounced at the end, than at the beginning -arrows)

We now implement contractions causing neck flexure. The contraction along the ventral area models the effect of the heart purse-string pull [9, 30]. Indeed during early embryo morphogenesis one anterior sector constricts to form a ventral “purse-string” which contributes to shaping the heart territory, Video 14 shows the dynamics of contraction of the ventral purse string [9]. One effect of this constriction is to flex the head ventrally. Figure 10a shows a profile view of chicken embryo morphogenesis during this event, showing the anterior flexure, as the ventral tissue constricts (see Video 15). We can implement a ventral pull, by ascribing a shearing force to the inferior edge of the calculation. This gives typical neck flexures as in Fig. 10b, which accompanies the ear formation (Video 16). This model reproduces at a qualitative level the ventral flexure of the neck area, while the ear and mouth are simultaneously formed. We observe (Fig. 11) that the ventral pull tends to delay the invagination of the mouth by antagonizing the inwards deformation of the mouth.

**Fig. 12:**
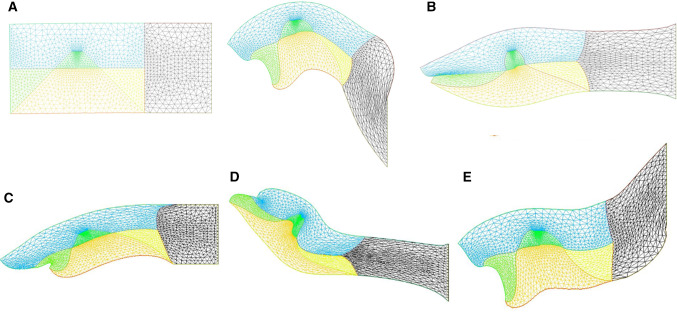
**a** Combination of neural extension, mouth contraction and ventral contraction, generating a quite flexed head with an important neck curvature. The ventral contraction hinders the mouth invagination. We see that the shape of the lower and upper jaws are different because of the different traction forces. **b** Combination of neural extension, mouth tension, and moderate ventral contraction giving a more elongated pattern, a flatter head with little flexure, and a more pronounced mouth invagination. **c** Combination of neural extension and mouth contraction, giving and inward rotation and invagination of the mouth. Video 17. **d** Combination of asymmetrical mouth contraction and DV contraction. The asymmetry of mouth contraction generates an asymmetry in upper vs lower jaws. **e** Combination of dorsal extension, mouth contraction, and ventral contraction, provoking an inversion of curvature between the head and the neck

However, the embryo may also constrict along the midline, since, during embryogenesis the crest of the neural tube corresponds to one of the rings. We then simply add a shearing force along the dorsal segment of the rectangle, which now causes an upward bending of the embryo body (Fig. 12). We see that it is quite easy to generate heads with different curvatures of necks, as classically observed, by unequal shearing forces exerted ventrally or dorsally.

**Fig. 13:**
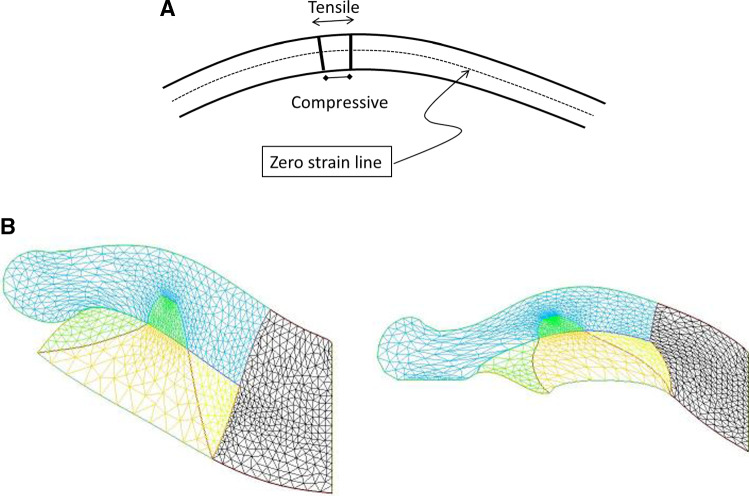
**a** When a thin shell is bent, the outer part is in a tensile state and the internal part in a compressive state. We assume that cells respond to the strain by growth, which adds a negative surface tension in the cell layer. **b** Left, a nasal process grown with a constant negative surface tension (Video 19). Right, a nasal process grown with a negative surface tension proportional to the local curvature (Video 21). The process is nonlinear, and it tends to generate elongated features, with flatter sides

### Implementation of extensional shear forces due to growth

We considered above only tension-driven contractions along the set of orthogonal lines. We argued that such contractions self-arrest. Now, returning to actual embryogenesis, we learn that embryos progressively extend along the axis corresponding to the folded rings (tubes), especially along the neural tube, which is located dorsally. This is a secondary phenomenon. While this extension mechanism is in itself a matter of fundamental research in biology [21], from a topological point of view it amounts to a transposition of forces around hyperbolic points during neurulation, by transposition we mean a swap of axis XX’ and YY’. Indeed, as the embryo constricts in the medio-lateral direction, it flattens in the other. The transposition along the A-P axis of the tissue results in an extension force along that axis.

We can return to our model and add now an extension along the dorsal line to include the effect of neural tube elongation. This extension can be written as a negative shearing force, oriented outwardly instead of converging. This will tend to increase dimension instead of shrinking it, and cause an extension of facial features. This tends to generate elongated heads as in Fig. 12 Video 17. We give a few examples of different forms obtained by playing with the parameters in Fig. 12.

### Implementation of a negative surface tension

In the previous part, we implemented an extensional gradient of tension by shearing forces oriented outwardly. However, one can model formally extensional growth as a negative surface tension. Indeed, when cells are stacked parallel in a ridge and grow, they exert a compressive growth stress oriented sideways. This sideway force behaves as a negative contribution to tension [33]. Negative surface tension is familiar in electrochemistry where it derives from electric interaction between charges. This is well known to cause the Lippman electrocapillary effect [34]. For the potential of zero charge, the surface tension exhibits a maximum. Charging negatively or positively an interface adds a negative component to the surface tension since charges of the same sign tend to repel each other, causing a decrease in surface tension equivalent to a compressive stress [35]. This effect is qualitatively similar to the effect of mechanotransduction, which causes a reduction in tension under either compressive or tensile stress, by growth. While this had been hypothesized theoretically in Ref. [33], it was observed recently that artificial compression and bending of epithelia causes a relaxation of actin-myosin pre-stressed state, which induces flattening of the tissue [36]. Also, it has long been known in plant biology that bending induces growth and other types of feedback (flowering) [37].

Negative surface tension, by definition, favors growth since it spontaneously works to increase the area. This is different from, say, Saffman–Taylor fingering [38], in which the positive surface tension opposes extension forces, and the S-T fingers grows only thanks to the internal pressure which works against the viscous drag and the surface tension. Here, surface growth will suffice to extend the pattern. The negative surface tension in biological tissue does not derive from a thermodynamic potential, but from a mechanical stress (cells push to divide and insert new cells). We have shown in Ref. [33], with a 1D model, that tissue growth may be able to propagate a Lippman-type buckling instability in biological tissue. Here, we wish to implement a negative surface tension in our model.

It first proved necessary to smoothen the corner of the head, since the curvature is not defined at a sharp corner (Fig. 13a). We implemented a negative surface tension in the external contour only, because Dapogny’s algorithm only works for the external boundary of the domain. Technically, the negative surface tension is implemented in FE by introducing a normal growth stress in the boundary stress continuity term proportional to the curvature $\upkappa$. We find, for negative surface tension in the top of the mouth, the spontaneous extension of a roundish lip, nasal process or gum, as often observed in animals (Fig. 13 Left, Video 18).

However, the growth dynamics itself is known to be dependent on the stress, and not a constant, so the negative surface tension imparted by cell should not be a constant. Laws of mechanics imply that cells in a curved surface see a tensile strain above the average line, and a compressive strain underneath, which are all the larger as the pattern is more curved [39]. In general terms, tensile stress is known to favor cell division [40], recent work shows that compressive stress also triggers proliferation [41]. Proliferation may be induced also indirectly by regulation of growth factor receptors instead of the growth factors themselves, as for example in epidermis growth during formation of epidermal ridges which show an upregulation of the receptor EGFR in the more curved areas of the dermis (see Fig. 4d in Ref. [42]). If we assume that cells react in proportion to their strain, we see that the negative component of the surface tension will be proportional to curvature: more curved areas will tend to grow faster, as seen for example in sweat ducts [42]. This renders the growth dynamics quadratic in curvature, since the magnitude of the out-of-plane force will be proportional to curvature by the Laplace formula (at more curved locations, the growth is faster because tension is more negative, but since the curvature is higher there, the normal component of the growth is higher too by a factor $\upkappa$, hence a total effect of order $\upkappa ^{2})$. If we implement a normal growth which is proportional the square of the curvature $\upkappa$, we find more elongated nasal processes, evoking elephant trunks and the like (Fig. 13 Right Video 19). We suggest that this effect may explain the expansion of edges such as nares, ear pavilions, eye lids etc.

**Fig. 14:**
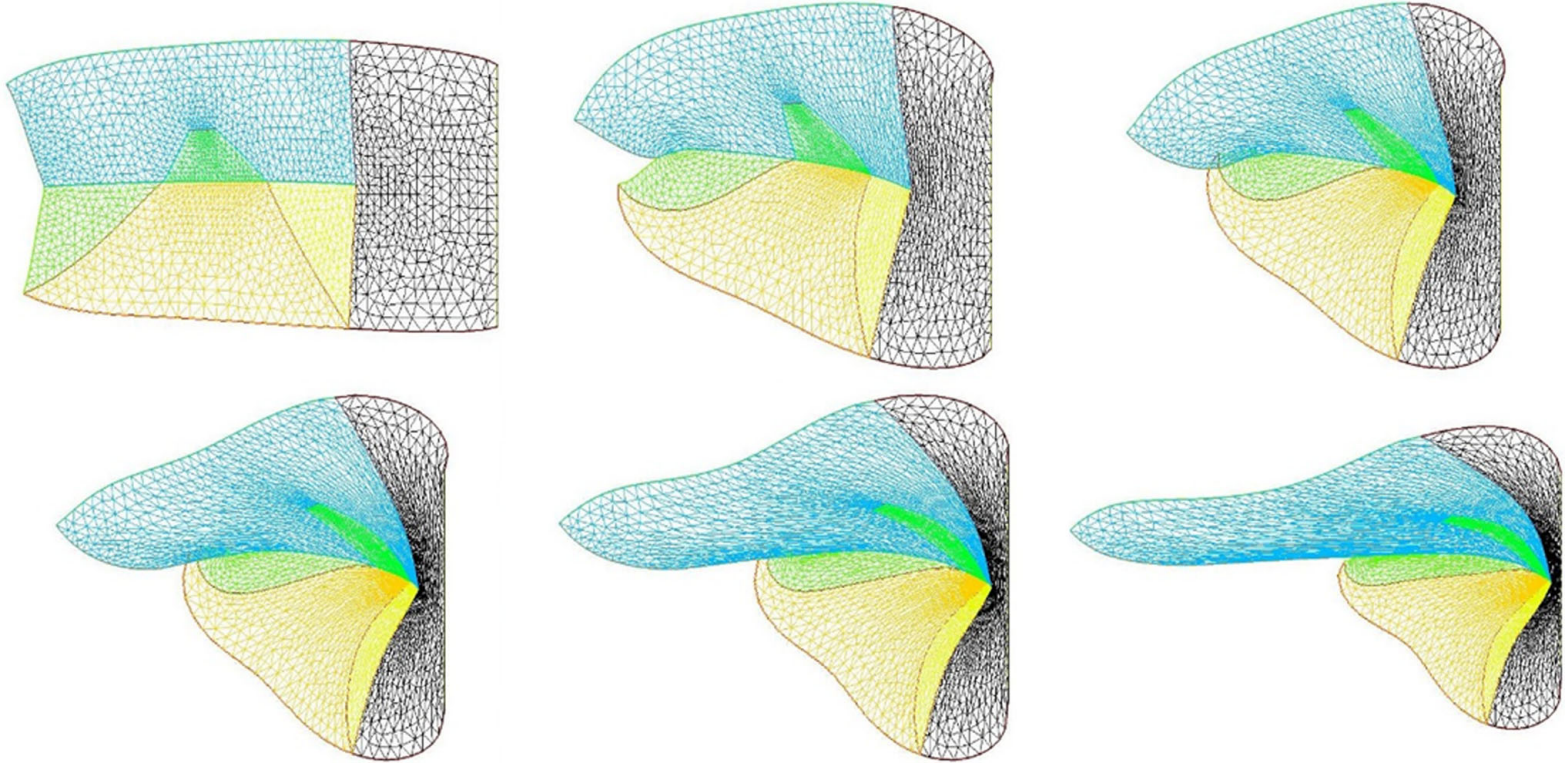
If we assume that the normal surface tension, and the surface shear are free variables, we can get a wealth of forms including elephant-like patterns. (Video 20),

**Fig. 15:**
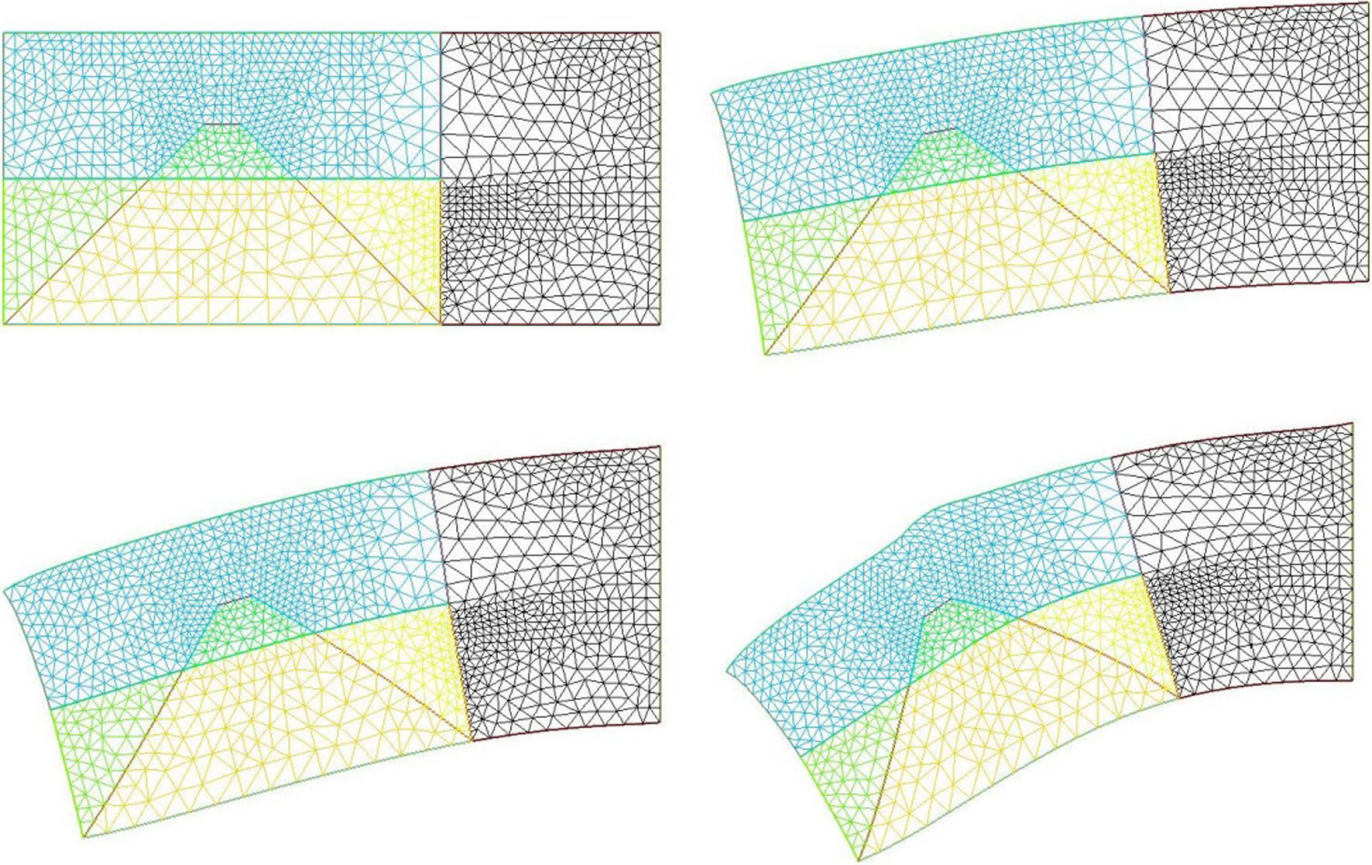
A deterministic buckling can be obtained with negative surface tension causing a bulge to form above the trapeze of the future sensory organ (say an eye) (Video 21)

### Relaxing all parameters

When the magnitude of the tension varies with the curvature, it varies with the position. So the variation with curvature induces also a surface shearing force due to the gradient of surface tension. The surface shearing force is oriented from the low tension to the higher tension (the higher tension pulls more), so the gradient of tension will tend to flatten the surface. We see that this situation is the opposite as the one explained earlier. In paragraph II.3 above, we had an initially flat mouth, in which the shearing component of the tension was opposed by the normal component, which tended to flatten the surface. Now in case of a negative surface tension, the normal component is oriented outwardly, but the gradient of curvature (hence the gradient of tension) tends to flatten the surface. Therefore, the simulation in Fig. 13 Right is an extremal case, neglecting the surface shearing component. While the present technical state of the Freefem$++$ software does not make it possible to introduce a surface shear varying with the gradient of curvature, we nevertheless can relax the relationship between tension gradient and surface curvature and implement in our model a normal stress in addition to a tangent shear with free parameters for the two stresses (normal and tangent) at the boundary. This is able to give such forms as the “elephant” in Fig. 14 (Video 20).

### Buckling instability

When the parameters are driven to higher values, the model generates buckling instabilities. These were predicted in Ref. [33], and hypothesized to be related to somite formation. We shall not go into a detailed study of buckling here. However, one surprising result is worth mentioning. When implementing a negative surface tension effect proportional to $\upkappa$ along the top side of the “head”, and simultaneously a small winding of the trapeze territory forming our ear or eye “placode”, we observed that the very small movement of the trapeze oriented “towards the top” in our geometry, would suffice to nucleate a surface buckling forming a bump swerving around the trapeze (Fig. 15, Video 21). This means that the bulge is not a direct deformation induced by the sensory organ but an indirect one by stimulating the growth geometrically. This may well describe the formation of a thick superorbital bulge or ridge around the eyes. This shows that a small localized strain may induce a deterministic bulge on the surface, by a negative surface tension implied by growth.

## Conclusion

In the end, this simple model is able to form different patterns which arguably, are reminiscent of what we see in animals. Animals are described here as the concatenation of three physical phenomena: minimal cleavage, followed by one first folding along the orthoradial lines of that cleavage, and a secondary folding along the deformed radial lines. This renders animal formation amenable to a general mechanistic law. While we played here with quite many parameters, in the real world acto-myosin contraction in different parts should be in similar magnitude ranges.

We acknowledge that a simple 2D model representing a lateral projection of a cylindrical tube is a very crude description of a tubular animal. However, the model finds several features which are observed in animals. Especially, we observe that subsequent contractions tend to slow down and even in some cases arrest themselves spontaneously. In this view, an animal is a scar. Actually, the initial distribution of stress is physically frustrated or unbalanced in the reference configuration. The situation is not physically compatible with immobility. Embryogenesis appears here as a dynamic process deforming self-consistently the reference configuration and the source terms within it, towards a less deformable form in which contraction forces are congruent with the form viewed as a simultaneous geometrical and dynamical fixed point.

We observe that invagination, such as mouth invagination, may occur progressively by a continuous visco-elastic movement under tension gradients, without requiring actual buckling (no threshold). The contractions tend to remodel sectors into round orifices, or slits. Asymmetrical contractions either Dorso-Ventral or Antero-Posterior, tend to transform orifices into “ ear-like ” patterns. More complex forms are associated with head flexure. Shear forces with a push-pull action of the neural tube on top and of the Dorso-Ventral boundary and heart territory along the A-P axis flex more or less the neck, in the typical pattern seen in necks.

The model highlights complex non-intuitive correlations between the deformations, which are constrained by laws of visco-elasticity. The long range nature of mechanical deformations correlates the morphogenesis of different parts: neck flexure influences mouth development, ear development is asymmetrical due to the asymmetry of ventral flexure, sensory organs tend to stimulate the formation of a bulge around them, etc. This gives some mechanistic support to the “mysterious laws of correlation” between body parts during evolution, as was observed and commented by Charles Darwin^3^ [43].

Cell stacking in extending elements plays an important role by inducing the equivalent of a negative surface tension. The effect of cell stacking is found to be nonlinear, and to vary as the square of the curvature, which may explain why animals tend progressively to show elongated slender features, instead of being a mere grape of round vesicles, as observed at early embryonic stages. We suggest that this phenomenon is at play in the natural tendency of edges or ridges to run away and form elongated tissue flaps such as ear pavilions, or trunks.

Despite the biological complexity of animal development, this simple model with a limited number of parameters, may help heuristically to relate seemingly unrelated forms which appear at first glance very different, but can actually be obtained one from another, by changing simply the magnitude of a few parameters, acting like cursors. This work shows that one aspect of the dynamics of embryo formation is fundamentally biaxial. Biaxiality is found at different scales : at a molecular scale, the actin-myosin complexes assemble into sarcomeres which have a biaxial stacking [44]. Cells along ridges such as the neural crest or the ear contour align radially and stack orthoradially, as seen, for example, in Fig. 4. Oocyte cleavage is bi-axial, and finally, at long range, there exists in embryonic tissues a radial and orthoradial texture inherited from early embryo cleavages. This provides a model for the force terms in between the small and the large scale, in the spirit of Ben Amar et al. [45]. The calculations are very robust, since they are based on a quasi-static approximation, which is justified by the slow pace of embryo development. However, the calculations assume a reference configuration, in which the orthoradial contraction forming the Antero-Posterior tubes occurred sooner than the radial contractions, causing the formation of the sensory organs. One may wonder what would occur if the order of the contractions were opposite. The concepts put forward here would predict a completely different plan with perforated radial tubes converging towards a center.

The approach described here is fundamentally different from a biochemical approach *à la Turing*, of face formation. In the reaction–diffusion description of sensory organs each organ precursor of the face is a standing maximum of chemicals, which may have an arbitrary position related to scalar gradients of chemicals and *ad hoc* biochemical feedback loops. The biaxial, mechanical, approach relates instead the position of the sensory organs to a long range checkerboard pattern, or rather darts target pattern, present in the blastula, whose origin is in the dichotomous nature of cell division, and the mechanical deformations which it implies. Since cell-division is bi-axial and since it progresses in a top-down scaling orientation, the presence of orifices oriented towards the neural tube is a consequence of the tensorial nature of embryogenesis. In this view, the existence of sensory organs, and ultimately of a face, is a remote but fundamental consequence of cell cleavage.

From an artistic perspective, the presence of a biaxial set of force actuators, with reduced scalar parameters acting as cursors, may help to morph simply anatomic patterns in the context of 3D animation [46].

## Supplementary Information

Below is the link to the electronic supplementary material.

Supplementary file 1 (avi 21606 KB)

Supplementary file 2 (avi 18558 KB)

Supplementary file 3 (avi 9184 KB)

Supplementary file 4 (avi 7714 KB)

Supplementary file 5 (avi 30785 KB)

Supplementary file 6 (avi 11281 KB)

Supplementary file 7 (avi 13235 KB)

Supplementary file 8 (avi 4667 KB)

Supplementary file 9 (avi 4169 KB)

Supplementary file 10 (avi 8116 KB)

Supplementary file 11 (avi 20513 KB)

Supplementary file 12 (avi 9683 KB)

Supplementary file 13 (avi 7024 KB)

Supplementary file 14 (avi 17914 KB)

Supplementary file 15 (avi 18185 KB)

Supplementary file 16 (avi 5908 KB)

Supplementary file 17 (avi 10777 KB)

Supplementary file 18 (avi 10809 KB)

Supplementary file 19 (avi 9814 KB)

Supplementary file 20 (avi 10144 KB)

Supplementary file 21 (avi 10174 KB)

Supplementary file 22 (pdf 659 KB)

## Footnotes

1 They are not strictly radial in that they have to reconnect at right angles in the central part.

2 Are not strictly radial in that they have to reconnect at right angles in the central part (Fig. 2, left). In French : développement *vs* croissance.

3 “If man goes on selecting, and thus augmenting, any peculiarity, he will almost certainly modify unintentionally other parts of the structure, owing to the mysterious laws of correlation”, Chapter I, paragraph 2 “effects of habit and of the use or disuse of parts ; correlated variation; inheritance [43].
