# Correction to: A biaxial tensional model for early vertebrate morphogenesis

**DOI:** 10.1140/epje/s10189-022-00199-x

**Published:** 2022-05-12

**Authors:** Vincent Fleury, Anick Abourachid

**Affiliations:** 1 Laboratoire MSC, CNRS/Universit é de Paris Cité, UMR 7057, 10 rue Alice Domont et Ĺeonie Duquet, 75013 Paris, France; 2 Laboratoire Mécanismes Adaptatifs et Evolution, UMR 7179 MNHN/CNRS, CP 55, 57 rue Cuvier, 75231 Paris Cedex 05, France

## Correction to: Eur. Phys. J. E (2022) 45:31 10.1140/epje/s10189-022-00184-4

Figure 1b is missing from the pdf version of this article; the figure should have appeared as shown below. The original article has been corrected.

**Fig. 1:**
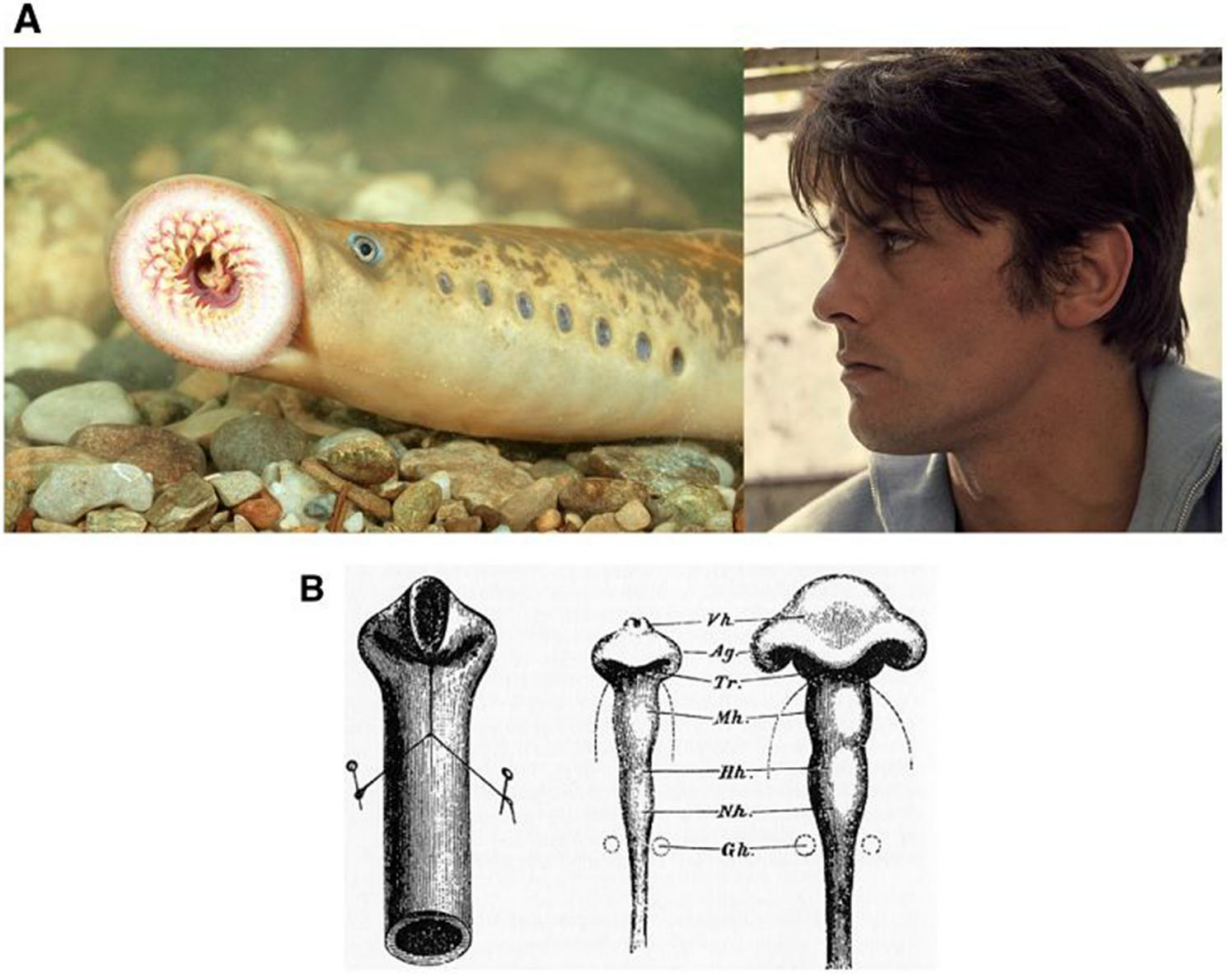
**a** To the left, the lamprey or “flute-fish” exhibits a striking structure composed of a cylindrical body, having cylindrical “holes” with translational symmetry along the dorso-ventral boundary, and one large anterior circle (the mouth), located around the axis of symmetry. To the right, a typical human profile. In higher vertebrates, head flexure locates the mouth, nose, eyes and ears along a deformed anterior tube. **b** The flexure of the neural tube, as invoked by Wilhelm His (1831–1904). The flexure of the neural tube (Right), is supposed to be analogous to the flexure of a rubber tube (Left) (©Michael Holtz/Photo12)

