# Supplementary material for: A biaxial tensional model for early vertebrate morphogenesis
Source: Eur Phys J E Soft Matter. 2022 Apr 8;45(4):31. doi: 10.1140/epje/s10189-022-00184-4 (PMC8993754; doi:10.1140/epje/s10189-022-00184-4)
Supplement: Supplementary file 22 — (pdf 659 KB) [file 10189_2022_184_MOESM22_ESM.pdf]

Supplementary Material.

Methods

Methods 1. Embryo time-lapse imaging

The experimental procedure to obtain the time-lapse imaging of embryo development has been described in Refs. 17 and 18. All experiments observations reported here occur during the first 4 days of development (the latest in this series being the closure of the amniotic sac). Embryos are observed shell-less in custom petri-dishes designed to have a small enough height to accommodate the heating stage from Minitüb gmbh) under the microscope (either a Leica MacroFluo or a Nikon Eclipse).

To be noted, the observation of embryos in profile view requires either to orient the embryo with tweezers in a horizontal profile view, or to use a microscope which is installed horizontally.

The observation of a normal ear development requires preparing embryos after neurulation, otherwise ear formation does not proceed normally (after neurulation, the embryonic body forms a stiff A-P tube which is less sensitive to the surrounding stresses; before neurulation, the folding embryo is quite sensitive to stress relaxation when it is cut off from the vitelline membrane and transferred to shell-less mode). There is a delay of about 2 hours between the development of the left and right ear, which compensates for the natural variability of embryo development ; the fact that there is a temporal difference between L and R ear development allows one to select the best ear for T-L imaging.

Images are acquired without any staining. To get an acceptable contrast one needs to rinse off carefully all the yolk, and to use a double-slit cache, for the light, which works as a Mac-Zender type interferometer (the light diffracted by one slit interferes with the direct light causing an enhancement of contrast).

Methods 2. Numerical simulation

Freefem++ code for generation of the images in this article.

The main parameters are :

-the mesh structure, which is defined by a number of edges, corresponding to all the segments visible in the simulation (including the boundary conditions). These segments receive different labels.

-the values of the contraction or extension parameters along each segment taking care also of the definition of the Normal vector, which depends on the orientation of the segment.

3-the value of the negative surface tension, if any.

-the boundary condition, at least one point somewhere must be fixed (here generally the segment to the right).

The surface gradient of tension term is written as a shearing force $\alpha\mathbf{T}$ along the tangent vector. This implies introducing a term α(Tx.s+Ty.w) in the boundary integral in the weak formulation of the Finite Elements, with Tx=Ny and Ty=-Nx. A growth force corresponding to a gradient of surface stress is defined by a boundary term α(Nx.s+ Ny.w) ; a negative tension γ representing the effet of lateral ectodermal expansion is written as a surface pressure $\gamma\mathbf{N}\kappa$ normal to the surface, and proportional to the local curvature κ. The "curvature" module of Dapogny et al.[37] is used to extract the value of κ from the mesh. If the growth kinetics is dependent on curvature, then we add a term $\mathbf{N}\kappa^2$ in the

boundary integral of the Finite Element formulation. We give here after one typical code, which can be copy-pasted by anyone wishing to reproduce the results in this article.

The edges names in the simulation are defined as :

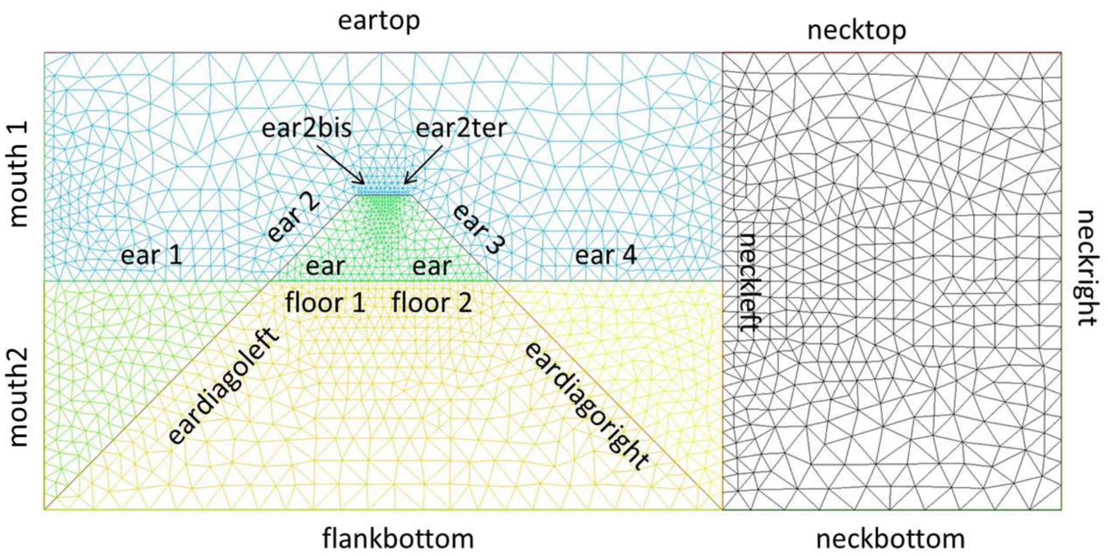

Supp. Material Example of code

Example of code without the curvature module
Gives the head shown in Fig. 12 A

```
//  Visco morphogenesis of an embryo from biaxial tensions
//   It uses a step by step Hookean-lke (generalized Netonian) model as
constitutive equation
//  Tension is along a pattern of lines inherited from neurulation
//  Simulation of head deformation under biaxial tractions in the mouth
area and the ear area
//  Uses the Freefem++ pakage

border ear1(t=0,0.2){x=-0.1+t;    y=0.0;  label=6;};
border ear2(t=0,0.075){x=0.1+t;    y=0.0+t;  label=1;};
border ear2bis(t=0,0.025){x=0.175+t;    y=0.075;  label=1;};
border ear2ter(t=0,0.025){x=0.200+t;    y=0.075;  label=1;};
border ear3(t=0,0.075){x=0.225+t;    y=0.075-t;  label=1;};
border ear4(t=0,0.2){x=0.3+t;    y=0.0;  label=3;};
border earRight(t=0,0.2){x=0.5;    y=0.0+t;  label=1;};
border earTop(t=0,0.6){x=0.5-t;    y=0.2;  label=1;};
border mouth1(t=0,0.2){x=-0.1;    y=0.2-t;  label=4;};
border flankRight(t=0,0.2){x=0.5;    y=0.0-t;  label=1;};
border flankBottom(t=0,0.6){x=0.5-t;    y=-0.2;  label=7;};
border mouth2(t=0,0.2){x=-0.1;    y=-0.2+t;  label=2;};
border eardiagoleft(t=0,0.2){x=0.1-t;    y=0.0-t;  label=1;};
border eardiagoright(t=0,0.2){x=0.3+t;    y=0.0-t;  label=1;};
border earfloor1(t=0,0.1){x=0.3-t;    y=0.0;  label=9;};
border earfloor2(t=0,0.1){x=0.2-t;    y=0.0;  label=10;};
border neckventral(t=0,0.2){x=0.8;    y=-0.2+t;  label=5;};
```

```
border neckdorsal(t=0,0.2){x=0.8;   y=0.0+t;    label=5;};
border neckBottom(t=0,0.3){x=0.5+t;   y=-0.2;  label=8;};
border neckTop(t=0,0.3){x=0.8-t;   y=0.2;  label=1;};
border neckMidline(t=0,0.3){x=0.5+t;   y=0.0;  label=1;};

mesh      th      =      buildmesh(ear1(15)+earfloor2(-15)+earfloor1(-
15)+ear4(15)+earRight(15)+earTop(20)+mouth1(20)+ear2(-10)+ear2bis(-10)
+ear2ter(-10)+ear3(-10)+eardiagoleft(-20)+mouth2(-
15)+eardiagoright(20)+flankRight(-15)+flankBottom(-
25)+neckBottom(10)+neckventral(10)+neckdorsal(10)+neckTop(10));

plot(th,wait=0);
//+flankRight(-15)+flankBottom(-25)) ;
//+flankRight(-15)+flankBottom(-25)+flankLeft(-15));

real E = 2.5;
real sigma = 0.29;
real mu = E/(2*(1+sigma));
real lambda = E*sigma/((1+sigma)*(1-2*sigma));
real gravity = -0.00;

real sigma11=0;
real sigma22=0;
real sigma16=0.0200;
real sigma13=-0.02000;
real sigma14=0.0082500;
real sigma12=-0.00825000;
real sigma17=0.00025000;
real sigma18=-0.00025000;
real sigma19=0.0101;
real sigma110=-0.01001;
real coef=3;

//mesh th = buildmesh( b(20)+c(5)+d(20)+a(5));

fespace Vh(th,[P1,P1]);
Vh [uu,vv], [w,s];
cout << "lambda,mu,gravity ="<<lambda<< " " << mu << " " << gravity <<
endl;
// deformation of a beam under its own weight
// definition of 2 macro :
real sqrt2=sqrt(2.);
macro epsilon(u1,u2)  [dx(u1),dy(u2),(dy(u1)+dx(u2))/sqrt2] // EOM
macro div(u,v) ( dx(u)+dy(v) ) // EOM

int i,j;

for (i=0;i< 500;i++)

{

for (j=0;j< 5;j++)

{solve bb([uu,vv],[w,s],solver=CG)=
      int2d(th)(
              lambda*div(w,s)*div(uu,vv)
                   +2.*mu*( epsilon(w,s)'*epsilon(uu,vv) )
                  )
```

```
  - int2d(th) (gravity*s)

+    int1d(th,6)(coef*(sigma16*(N.y*w-N.x*s) ) )
+    int1d(th,3)(coef*(sigma13*(N.y*w-N.x*s) ) )

+    int1d(th,7)(coef*(sigma17*(N.y*w-N.x*s) ) )

+    int1d(th,2)(coef*(sigma12*(N.y*w-N.x*s) ) )
+    int1d(th,4)(coef*(sigma14*(N.y*w-N.x*s) ) )

+    int1d(th,8)(coef*(sigma18*(N.y*w-N.x*s) ))
+    int1d(th,9)(coef*(sigma19*(N.y*w-N.x*s) ))
+    int1d(th,10)(coef*(sigma110*(N.y*w-N.x*s) )   )

+ on(5,uu=0,vv=0)

;

mesh th1 = movemesh(th, [x+0.5*uu, y+0.5*vv]);

plot(th,wait=0);

//plot(ps="maillage.eps");

cout << " max deplacement = " << uu[].linfty << endl;
th=th1;
}
plot(th,wait=0);

}
```

Supp. Material Videos

Video 1 Early stage of Chicken embryo neurulation, Mag. 4X. One sees the kinks in the neural fold corresponding to the locking of the ocular territory.

Video 2. Formation of ear and eyes in a chicken embry (low resolution, Leica Macro-fluo Binocular). The eye forms while the ear is already formed. The closure of the amniotc sac is also visible.

Video 3 Formation of the nasal pit in a chicken embryo. (Mag 4X, Nikon Eclipse microscope). The nasal pit forms while the eye is already formed.

Video 4 Closure of a circular hole under the tension at the edge (linear shrinking).

Video 5 Time-Lapse video-microscopy of amniotic sac closure (chicken embryo, Mag. 0.8X).

Video 6 Contraction of a DV boundary causing the transformation of a crenel territory into a pear-like domain (exponential slowing down).

Video 7 Time-Lapse video-microscopy of ear formation (chicken embryo, Nikon Eclipse Microscope Mag. 10X). (Video part of Supp. Material of Ref. 19).

Video 8 Contraction of a DV boundary, plus tangent shear force oriented towards the “top” in the simulation, following the edge of the trapeze. This causes the rounding off a crenel territory.

Video 9 Asymmetrical contraction of a DV boundary, plus asymmetrical tangent shear force oriented towards the “top” in the simulation, following the edge of the trapeze. This causes an asymmetry leading to a form reminiscent of an ear contour.

Video 10 Contraction of a DV boundary causing the formation of a slit. In the absence of tissue in the hole, or if the tissue is buckled or very soft, the pattern will collapse into the shape of a slit.

Video 11 Time-Lapse video-microscopy of mouth formation (chicken embryo, Mag. 10X). This is the earliest stage of mouth formation, when the anterior sector starts to constrict. The anterior oral sector, intersected by rings, constricts, and the mouth territory progressively rounds off. The movement is in plane. (Video part of Supp. Material of Ref. 19).

Video 12 Time-Lapse video-microscopy of mouth formation at the stage after neurulation (chicken embryo, Mag. 4X). The head rocks and flexes forward, the edges of the mouth territory constrict and the gums start to form.

Video 13 Simulation of simultaneous mouth invagination and ear contraction.

Video 14 Time-Lapse video-microscopy (underneath view Mag. 4X) of the ventral contraction. The film shows the formation and constriction of the cardiac purse-string, which pulls on the head features. The head parts are visible in the background, across the blastodisc tissue. This ventral constriction results in a “downwards”, ventral flexure of the entire head tissue.

Video 15 Time-Lapse video-microscopy (profile view 2X, then 4X) of head flexure under ventral contraction. As the anterior ring constricts, it passes underneath the embryo and constricts steadily. This ventral constriction results in a “downwards”, ventral flexure of the entire head tissue. (Video part of Supp. Material of Ref. 19).

Video 16 Simulation of head bending under ventral flexure, while the mouth invaginates.

Video 17 Simulation evoking a marine vertebrate, with an anterior extension of the dorsal part of the head (neural tube extension) leading to a ventral inwards roll up of the mouth.

Video 18 Simulation with a negative tension independent of curvature in the rostral area. A nasal process grows.

Video 19 Simulation with a negative tension proportional to curvature in the rostral area. A nasal process grows which is more elongated.

Video 20 Simulation of elongated nasal proeminence evoking trunk formation, obtained wth anisotropric extension of the dorsal and upper mouth area, and a normal feedback.

Video 21 Generation of a frontal superorbital bulge by negative surface tension buckling induced by the presence of the trapeze (ear or eye placode).
